# A New Basal Sauropodomorph (Dinosauria: Saurischia) from Quebrada del Barro Formation (Marayes-El Carrizal Basin), Northwestern Argentina

**DOI:** 10.1371/journal.pone.0026964

**Published:** 2011-11-09

**Authors:** Cecilia Apaldetti, Ricardo N. Martinez, Oscar A. Alcober, Diego Pol

**Affiliations:** 1 Museo de Ciencias Naturales, Universidad Nacional de San Juan, San Juan, Argentina; 2 Consejo Nacional de Investigaciones Científicas y Técnicas (CONICET), Argentina; 3 Museo Paleontológico Egidio Feruglio, Trelew, Argentina; College of the Holy Cross, United States of America

## Abstract

**Background:**

Argentinean basal sauropodomorphs are known by several specimens from different basins; Ischigualasto, El Tranquilo, and Mogna. The Argentinean record is diverse and includes some of the most primitive known sauropodomorphs such as *Panphagia* and *Chromogisaurus*, as well as more derived forms, including several massospondylids. Until now, the Massospondylidae were the group of basal sauropodomorphs most widely spread around Pangea with a record in almost all continents, mostly from the southern hemisphere, including the only record from Antarctica.

**Methodology/Principal Finding:**

We describe here a new basal sauropodomorph, *Leyesaurus marayensis* gen. et sp. nov., from the Quebrada del Barro Formation, an Upper Triassic-Lower Jurassic unit that crops out in northwestern Argentina. The new taxon is represented by a partial articulated skeleton that includes the skull, vertebral column, scapular and pelvic girdles, and hindlimb. *Leyesaurus* is diagnosed by a set of unique features, such as a sharply acute angle (50 degrees) formed by the ascending process of the maxilla and the alveolar margin, a straight ascending process of the maxilla with a longitudinal ridge on its lateral surface, noticeably bulging labial side of the maxillary teeth, greatly elongated cervical vertebrae, and proximal articular surface of metatarsal III that is shelf-like and medially deflected. Phylogenetic analysis recovers *Leyesaurus* as a basal sauropodomorph, sister taxon of *Adeopapposaurus* within the Massospondylidae. Moreover, the results suggest that massospondylids achieved a higher diversity than previously thought.

**Conclusions/Significance:**

Our phylogenetic results differ with respect to previous analyses by rejecting the massospondylid affinities of some taxa from the northern hemisphere (e.g., *Seitaad*, *Sarahsaurus*). As a result, the new taxon *Leyesaurus,* coupled with other recent discoveries, suggests that the diversity of massospondylids in the southern hemisphere was higher than in other regions of Pangea. Finally, the close affinities of *Leyesaurus* with the Lower Jurassic *Massospondylus* suggest a younger age for the Quebrada del Barro Formation than previously postulated.

## Introduction

Basal sauropodomorphs are non-sauropod sauropodomorphs that diversified and spread to most continents from the Late Triassic through the Early Jurassic, and constitute the first global radiation of herbivorous dinosaurs [1], [2]. In South America they are exclusively known from Brazil and Argentina. The Brazilian record includes *Saturnalia tupiniquim* [3]–[5] from the Carnian Santa Maria Formation; and the possible basal sauropodomorph *Guaibasaurus candelariensis* [6]–[8] and *Unaysaurus tolentinoi* [9] from the Carnian-Norian Caturrita Formation. The record from Argentina comprises *Eoraptor lunensis* [10], [11], *Panphagia protos* [12], and *Chromogisaurus novasi* [8] from the Carnian-Norian Ischigualasto Formation; *Riojasaurus incertus* [13], [14], *Coloradisaurus brevis* [15] and *Lessemsaurus sauropoides* [16], [17] from the Norian Los Colorados Formation; *Mussaurus patagonicus* [18], [19] from the Norian Laguna Colorada Formation; *Adeopapposaurus mognai* [20], [21] from the Lower Jurassic Cañón del Colorado Formation; and *Riojasaurus* sp. from the Norian? Quebrada del Barro Formation [22].

The assignation of the latter record—*Riojasaurus* sp—is questioned in this paper (see Age of Quebrada del Barro Formation), because the specimen is known only by a partial pes without any diagnostic features.

Herein we report a partial skeleton of a new basal sauropodomorph from the upper levels of the Quebrada del Barro Formation that has implications for our understanding on the diversity of massospondylid sauropodomorphs in South America and for the supposed age of this stratigraphic unit.

### Geological setting

**Figure 1:**
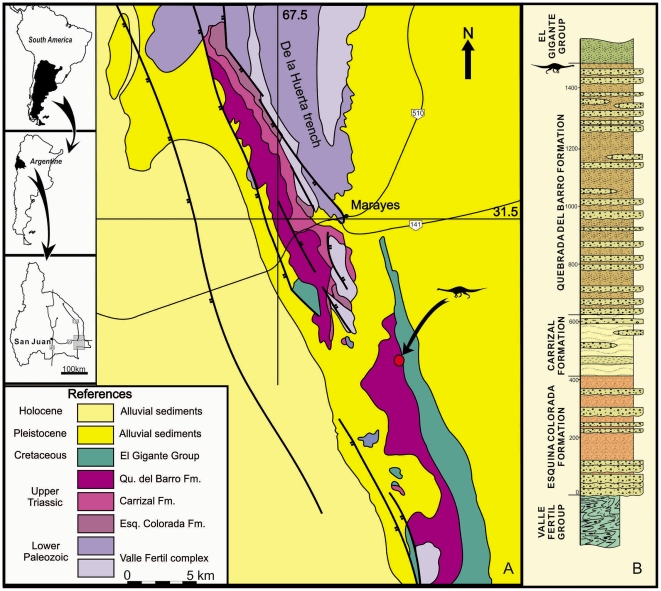
Location and Geologic map of the Marayes-El Carrizal Basin (Northwestern Argentina) . A: Location and geologic map; B: Section of the Marayes group at the type locality. The red circle indicates the site of the holotype of *Leyesaurus marayensis* gen. et sp. nov., near the top of the Quebrada del Barro Formation. (B: modified from Bossi [23]).

The Quebrada del Barro Formation (Marayes-El Carrizal Basin) crops out 140 km southeast of San Juan City, northwestern Argentina (Figure 1). This Formation is the uppermost unit of the Marayes Group [23]. The Marayes Group unconformably overlies lower Paleozoic metamorphic rocks, and is covered by remnants of the Upper Cretaceous El Gigante Group [24]. The thickness of the Marayes Group is 2300 meters and it is composed of three units: the Esquina Colorada, El Carrizal and Quebrada del Barro Formations (Figure 1B).

The Esquina Colorada Formation is composed of approximately 500 meters of conglomerates, sandstones, diamictites and ash layers [25], [26] (Figure 1B). Some fragments of vertebrate fossils were found in this unit, but these were not studied in detail [26]. The age of this unit was identified as to be Middle Triassic, correlated with Chañares, Ischichuca and Los Rastros Formations from the neighboring Ischigualasto-Villa Unión Basin [27].

The second unit is the El Carrizal Formation with 100–350 meters of sandstones, quartz conglomerates, siltstones and coal [26] (Figure 1B). This unit has provided flora and pollen that belong to the “*Dicroidium* flora”, suggesting a Late Triassic age by correlation with the Ischigualasto Formation from of the Ischigualasto-Villa Unión Basin [23].

The Quebrada del Barro Formation [26] tops the sequence with 900–1400 meters of reddish alluvial fan deposits that are mostly fine conglomerates and sandstones [23] (Figure 1B). The age of this unit was originally proposed as Cretaceous [28], [29], and later as Upper Triassic [23], [30]. In 1978, Bossi and Bonaparte [22] assigned a Norian age for Quebrada del Barro Formation based in the discovery of some vertebrate remains questionably assigned to *Riojasaurus* (see below).

## Methods

### Ethics statement

The Instituto and Museo de Ciencias Naturales has the appropriate permits needed to unearth the fossil materials that have been described in this paper (PVL 706).

### Preservation and preparation

The new specimen is a disarticulated incomplete skeleton. The proximity of all elements in an area of one half square meters, as well as the lack of duplicated elements and their relative size, suggest this association belongs to a single individual.

The specimen is well preserved and all the bones of the partial skeleton are three dimensional, complete, and most preserve fine anatomical details. The incompleteness of the skeleton is likely attributable to pre-burial processes.

The white bones of the holotype were embedded in a red fine-grained sandstone matrix with clay cement. The bones were prepared using a pneumatic air scribe pin vice, and water immersion.

### Terminology

We employ traditional anatomical and directional terms over veterinarian alternatives [31]. “Anterior” and “posterior”, for example, are used as directional terms rather than the veterinarian alternatives “rostral” or “cranial” and “caudal”.

We used different sources for phylogenetic definitions of taxa within Dinosauria: Sauropodomorpha [32], Anchisauria [2], Massospondylidae [33], Plateosauria [34], Eusauropoda and Neosauropoda [35].

### Source of comparative data

The comparisons with other sauropodomorphs and some theropods made in the description of the new specimen were based on the literature and on personal observation of specific taxa detailed in Table 1. Except in those cases where the source is specified, all the other references are based on the literature listed in this table.

**Table 1:** Sources of comparative data used in this study.

Taxon	Source(s)
*Adeopapposaurus mognai*	Martínez [21]; PVSJ 568, 569, 610
*Anchisaurus polyzelus*	Yates [42]; Fedak and Galton [53]
*Coloradisaurus brevis*	Bonaparte [15]; PVL 3967, 5904
*Efraasia minor*	Galton [54]; Yates [55]
*Eoraptor lunensis*	Sereno et al. [10]; Martinez et al. [11]; PVSJ 512
*Glacialisaurus hammeri*	Smith and Pol [45]
*Herrerasaurus ischigulastensis*	Sereno and Novas [56]; PVSJ 407
*Ignavusaurus rachelis*	Knoll [48]
*Jingshanosaurus* xinwaensis	Zhang and Yang [57]
*Lessemsaurus sauropoides*	Bonaparte [16]; Pol and Powell [17]
*Lufengosaurus huenei*	Young [58], [59]; Barret et al. [60]
*Massospondylus carinatus*	Cooper [44]: Attridge et al. [52], Sues et al. [61]
*Melanorosaurus readi*	Yates [34]
*Mussaurus patagonicus*	Pol and Powell [19]
*Panphagia protos*	Martinez and Alcober [12]; PVSJ 874
*Pantydraco caducus*	Yates [62]
*Plateosaurus engelhardti*	Galton [63], [64]; Moser [65]
*Riojasaurus incertus*	Bonaparte [13]; Bonaparte and Pumares [14]; PVL3803, PVSJ 849 (skull)
*Sanjuansaurus gordilloi*	Alcober and Martínez [66]; PVSJ 605
*Sarahsaurus aurifontanalis*	Rowe et al. [49]
*Saturnalia tupiniquim*	Langer [5]; Langer et al.[4]
*Seitaad ruessi*	Sertich and Loewen [47]
*Thecodontosaurus antiquus*	Benton et al [67]
*Unaysaurus tolentinoi*	Leal et al. [9]
*Yimenosaurus youngi*	Bai et al. [68]
*Yunnanosaurus huangi*	Young [69]; Barret et al. [70]
*Yunnanosaurus youngi*	Lu et al.[71]

All specimens compared in the text were observed from their respective source listed in this table. Comparisons based on other specimens, or taken from additional references, are explicitly indicated in the text.

### Nomenclatural Acts

The electronic version of this document does not represent a published work according to the International Code of Zoological Nomenclature (ICZN), and hence the nomenclatural acts contained in the electronic version are not available under that Code from the electronic edition. Therefore, a separate edition of this document was produced by a method that assures numerous identical and durable copies, and those copies were simultaneously obtainable (from the publication date noted on the first page of this article) for the purpose of providing a public and permanent scientific record, in accordance with Article 8.1 of the Code. The separate print-only edition is available on request from PLoS by sending a request to PLoS ONE, 1160 Battery Street, Suite 100, San Francisco, CA 94111, USA along with a check for $10 (to cover printing and postage) payable to “Public Library of Science”.

In addition, this published work and the nomenclatural acts it contains have been registered in ZooBank, the proposed online registration system for the ICZN. The ZooBank LSIDs (Life Science Identifiers) can be resolved and the associated information viewed through any standard web browser by appending the LSID to the prefix “http://zoobank.org/”. The LSID for this publication is: urn: lsid:zoobank.org:pub:66A4C4D7-E726-4C5B-8A0F-47FD9A109B01.

#### Institutional abbreviations

**BMNH**: Natural History Museum, London, UK. **BP**: Bernard Price Institute for Palaeontological Research, University of the Witwatersrand, Johannesburg; **BRSMG**: Bristol City Museum and Art Galleries, Bristol. **PVL**: Instituto Miguel Lillo, Tucumán, Argentina. **PVSJ**: Instituto y Museo de Ciencias Naturales, Universidad Nacional de San Juan, Argentina. **YPM**: Peabody Museum of Natural History, Yale University, New Haven.

## Results

### Systematic Paleontology

Dinosauria [36]

Saurischia [37]

Sauropodomorpha [38]

Massospondylidae [33]

### *Leyesaurus gen.* nov

urn:lsid:zoobank.org:act:3772093A-E5CD-48CF-9163-54E55FEDA240

#### Etymology

The generic name honors the Leyes family, inhabitants of the small town Balde de Leyes, who made the discovery and notified the paleontologists of the San Juan Museum.

#### Type Species

*Leyesaurus marayensis*

### *Leyesaurus marayensis sp*. nov

urn:lsid:zoobank.org:act:F7CD3D40-C64C-463E-A063-9E3FECF9C50D

#### Etymology

*marayensis* refers to the Marayes-El Carrizal Basin, where the specimen was found.

#### Holotype

**Figure 2:**
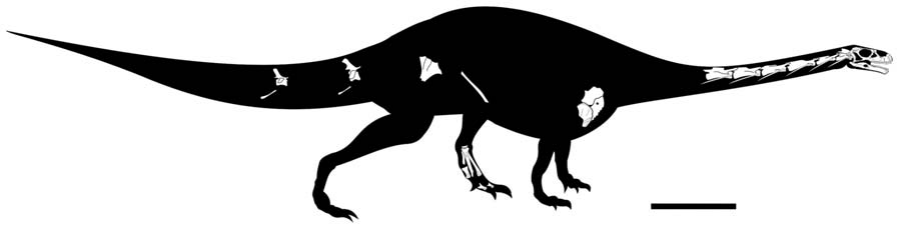
Silhouette reconstruction of the skeleton of *Leyesaurus marayensis* (PVSJ 706 ). Reconstruction only shows preserved bones. Modified from Martinez [21]. Scale bar equals 25 cm.

PVSJ 706, a partial skeleton including skull with articulated mandible, lacking both nasals, left prefrontal, middle section of the left maxilla, anterior half of the left lower jaw, supraoccipital, both exoccipitals, ophistotics, laterosphenoids and vomers; atlas-axis articulated with anterior cervical vertebrae (C3-C7); an anterior and a middle caudal vertebra; proximal region of the left scapula, coracoid and humerus; partial blade of the right pubis lacking distal and proximal ends; proximal region of both ischia; partial left pes that includs distal tarsals III and IV, metatarsal III lacking its distal end, complete metatarsals IV and V, first phalanx of digit I, second phalanx of digit II, and second phalanx of digit IV (Figure 2).

#### Type Locality

The type specimen was found near the locality Balde de Leyes, Caucete Department of San Juan Province, Northwestern Argentina (Figure 1A).

#### Horizon

The type specimen was found in red silty mudstones with a low clay cementation in the uppermost level of the Quebrada del Barro Formation [23], [26], 2 meters below the contact with the Cretaceous unit, Marayes-El Carrizal Basin.

#### Age of Quebrada del Barro Formation

The age of the Quebrada del Barro Formation was regarded as Norian by Bossi and Bonaparte [22] based on the presence of a single basal sauropodomorph specimen composed of an incomplete articulated right pes (including astragalus, calcaneum, two distal tarsals, metatarsal II, III, IV and V, and eight phalanges), and four incomplete caudal vertebrae. Based on the similarities between these materials and the basal sauropodomorph *Riojasaurus incertus* from the Norian Los Colorados Formation in the neighboring Ischigualasto-Villa Unión Basin, Bossi and Bonaparte [22] identified the specimen as *Riojasaurus* and proposed a Norian age for this unit [22]. Unfortunately, only the incomplete pes is housed at the Instituto Miguel Lillo, Tucumán, Argentina (PVL 4087) and the location of the caudal vertebrae mentioned by Bossi and Bonaparte [22] are currently unknown and precluding further analysis.

The lack of diagnostic characters in the PVL 4087 precludes its identification as *Riojasaurus incertus* (or any other basal sauropodomorph taxon). Since PVL 4087 lacks diagnostic characters, we interpret the specimen as a non-sauropod sauropodomorpha *incertae sedis*. Consequently, the rationale for the Norian age of the Quebrada del Barro Formation proposed by Bossi and Bonaparte [22] is pulled into question.

The close affinities of the new specimen reported here with the basal sauropodomorph *Adeopapposaurus* [21], as well as the close affinity of these two taxa with the Early Jurassic sauropodomorph *Massospondylus* from South Africa likely indicates a younger age for Quebrada del Barro Formation. *Massospondylus* is a typical vertebrate of the Lower Jurassic Upper Elliot and Clarens Formations in South Africa and the Forest Sandstone Group in Zimbabwe [39], [40], but is absent from the Late Triassic Lower Elliot Formation [41], therefore we suggest that the Quebrada del Barro Formation may be of Lower Jurassic age.

#### Diagnosis

A basal sauropodomorph diagnosed by the following autapomorphies and combination of characters (asterisks indicate autapomorphies): sharply acute angle (50°) formed by the ascending process of the maxilla with the alveolar margin*; straight ascending process of the maxilla with a longitudinal ridge on its lateral surface; noticeably bulging labial side of the maxillary teeth; greatly elongated cervical vertebra (length/height ratio of sixth cervical centrum more than 5*); neural arches of the cervical vertebrae with sinuous dorsal margin of the neural spines and short epipophyses—extending two-third of the length of the postzygapophyses—; and proximal articular surface of metatarsal III shelf-like and medially deflected*.

### Description

#### Skull roof

**Figure 3 pone-0026964-g003:**
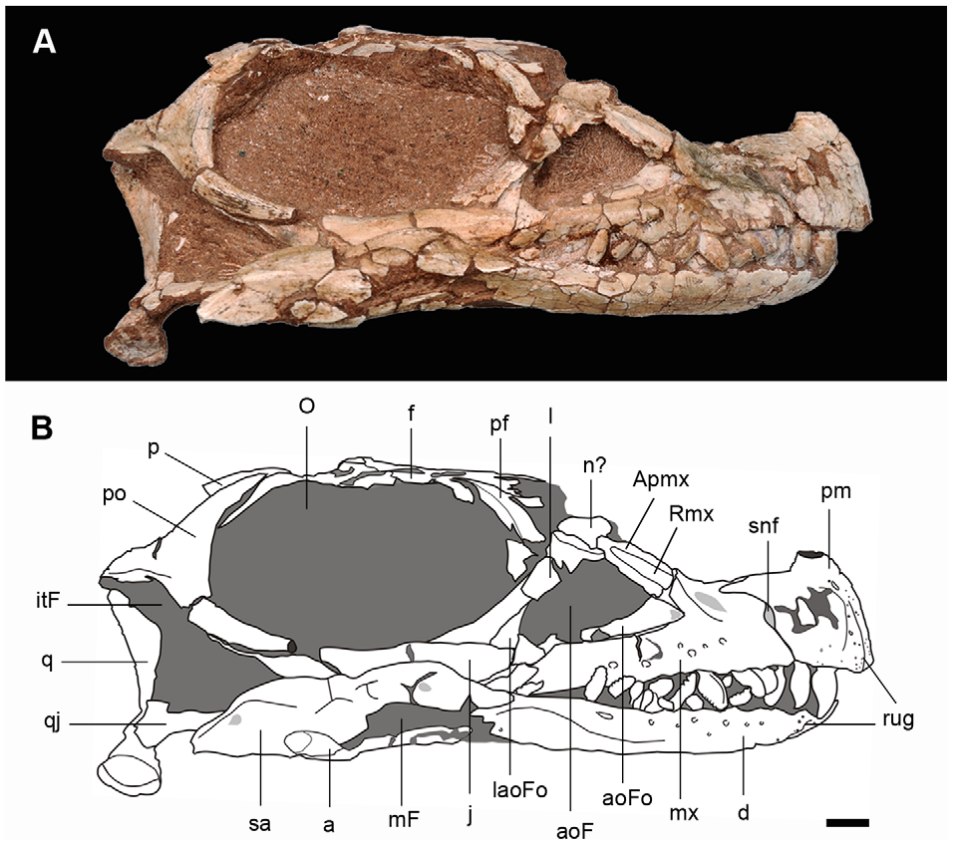
Skull of the new basal sauropodomorph *Leyesaurus marayensis* (PVSJ 706). Photograph of the skull (A) and interpretative drawing (B) in lateral view. Dark grey color represents matrix and light grey color represents foraminae. *Abbreviations*: *a*, angular; *aoF*, antorbital fenestra; *aoFo*; antorbital fossa; *Apmx*, ascending process of the maxilla; *d*, dentary; *f*, frontal; *itF*, infratemporal fenestra; *j*, jugal; *l*, lacrimal; *laoFo*; lacrimal antorbital fossa; *mF*, mandibular fenestra; *mx*, maxilla; *n*, nasal; *O*, orbit; *p*, parietal; *pf*, prefrontal; *pm*, premaxilla; *po*, postorbital; *rug*, platform-like rugosities; *q*, quadrate; *qj*, quadratejugal; *Rmx*, ridge of the ascending process of the maxilla; *sa*, surangular; *snf*, subnarial foramen. Scale bar equals 1cm.

The skull is relatively short and low (Figure 2, 3). Including the mandible, its height represents approximately 40% of the total length, although this may be an underestimate given the slight dorsoventral crushing of the specimen. Its maximum dorsoventral depth is in the anterior region of the postorbital, and its greatest transverse width is at the level of the sutural contact between the frontals and parietals (see Table S1 for measurements). The shape and size of the external naris cannot be inferred due to the lack of the nasals and dorsal processes of the premaxilla (Figure 3). The triangular antorbital fenestra is surrounded by a well-developed fossa along its anterior and ventral margins. The greatest anteroposterior length of the antorbital fenestra is approximately half the diameter of the orbit. The orbit extends over 30% of the total skull length, although this could be accentuated by the dorsoventral deformation (Figure 3). The infratemporal fenestra is hourglass-shaped in lateral view, with a dorsal end that is much narrower than the ventral end, which projects anteriorly below the orbit. The supratemporal fenestra has a subtriangular outline in dorsal view, and is slightly longer anteroposteriorly than it is transversely wide. This fenestra is also visible in lateral view, given that the posterior process of the postorbital lies below the dorsal rim of the orbit (Figure 3). Both external mandibular fenestrae are damaged and their margins have not been preserved.

**Figure 4:**
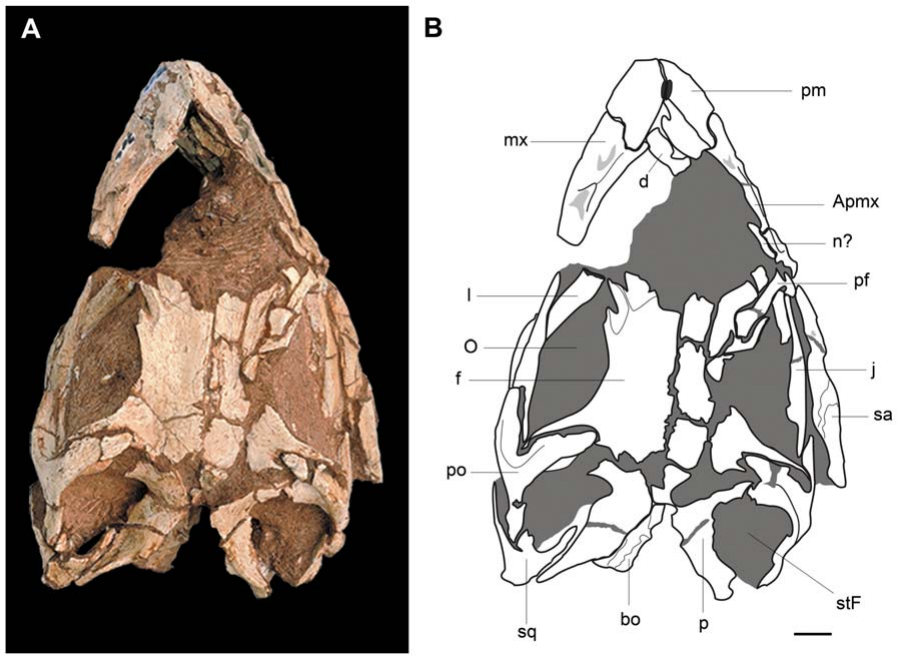
Skull of the new basal sauropodomorph *Leyesaurus marayensis* (PVSJ 706). Photograph of the skull (A) and interpretative drawing (B) in dorsal view. Dark grey color represents matrix and light grey color represents foramina. *Abbreviations*: *Apmx*, ascending process of the maxilla; *bo*, basioccipital; *d*, dentary; *f*, frontal; *j*, jugal; *l*, lacrimal; *mx*, maxilla; *n*, nasal; *O*, orbit; *p*, parietal; *pf*, prefrontal; *pm*, premaxilla; *po*, postorbital; *sa*, surangular; *stF*, supratemporal fenestra; *sq*, squamosal. Scale bar equals 1 cm.

Both premaxillae are preserved, but are lacking their dorsal processes (Figure 3, 4). In lateral view, the main body of the premaxilla is subquadrangular, as in *Adeopapposaurus* and *Massospondylus*. Its anterior margin is straight although its dorsal region is posterodorsally directed. Both premaxillae lack the dorsal processes and only preserve their bases, which are transversely compressed and oval in cross-section (Figure 4). The slender posterolateral process tapers backward and overlaps the dorsal edge of the anterior ramus of the maxilla. The posterolateral process and the posterior margin of the main body of the premaxilla form a right angle that produce an L-shaped suture between premaxilla and maxilla. An elliptical subnarial foramen is situated on the suture between the premaxilla and the maxilla. The anterolateral surface of the premaxilla has rugosities (Figure 3B) that form a similar platform to that of *Adeopapposaurus* and *Riojasaurus* (PVSJ 849). Moreover, this region is pierced by several small foramina scattered over the surface, the largest of which is located at the base of the dorsal process. In medial view, the premaxilla has a wide sulcus for the reception of the anteromedial process of the maxilla. *Leyesaurus* has a toothless gap between the first tooth and the symphysis. The gap or diastema has a length equals to the half of the anteroposterior length of the first dentary tooth.

The right maxilla is almost complete whereas the left element lacks its middle portion (Figure 3, 4). The maxilla has the typical triradiate morphology present in other sauropodomorphs. The anterior process of the maxilla contacts the premaxilla, the ascending process forms the anterior edge of the antorbital fenestra, and the posterior process extends along the ventral margin of the antorbital region (Figure 3). The length of the anterior process of the maxilla is anteroposteriorly shorter than its dorsoventral height, with parallel dorsal and ventral margins, as in *Massospondylus* and *Adeopapposaurus*. The ascending process is straight and posterodorsally oriented. The ascending process of the maxilla of *Leyesaurus* forms an angle of 50 degrees with the alveolar margin (Figure 3). This angle is more acute than the rest of non-eusauropod sauropodomorphs, in which the ascending process diverges from the alveolar margin of the maxilla at an angle ranging between 60 and 90 degrees (see Discussion). As in the massospondylids *Adeopapposaurus* and *Massospondylus* (SAM PK K1314; A. Yates, pers. com.), the ascending process of the maxilla is entirely straight, not posteriorly deflected at its dorsoventral end (where the nasal overlaps the maxilla), and differs from that of other basal sauropodomorphs in which the ascending process is posteriorly deflected along this region (see Discussion). In addition, the ascending process of the maxilla of *Leyesaurus* also differs from the rest of basal sauropodomorphs in that its lateral surface bears a rounded ridge that extends along its entire length (see Discussion). This ridge is broad at the base of ascending process and tapers dorsally (Figure 3). The posterior edge of the ascending process is sharp and forms the entire anterior border of the antorbital fenestra, as in *Adeopapposaurus.* The dorsal region of the ascending process is damaged and has not preserved its contact with the lacrimal. The posterior process of the maxilla forms the main body of this bone and tapers posteriorly. At least six neurovascular foramina are present on the lateral surface of the maxilla; most of them are aligned parallel to the alveolar border and open ventrally (Figure 3). The posterior end of the maxilla is crushed and is not possible to observe its contact with the jugal.

The ventral halves of the lacrimals are preserved (Figure 3), but both dorsal ends are damaged and the lacrimal foramen and its sutural contacts with the prefrontal, nasal, and ascending process of the maxilla cannot be determined. As in most sauropodomorphs, the main shaft of the lacrimal is anterodorsally inclined in lateral view (Figure 3). The lateral surface is straight, anteroposteriorly narrow, and its ventral portion is not as anteroposteriorly expanded as in *Plateosaurus*, *Anchisaurus*, *Massospondylus*, *Mussaurus*, *Unaysaurus*, and *Adeopapposaurus*. The antorbital fossa has a triangular shape and extends along the lower third of the total lacrimal height, as in other basal sauropodomorphs (e.g. *Plateosaurus*, *Massospondylus*, *Lufengosaurus, Adeopapposaurus*).

Only the right prefrontal has been partially preserved (Figure 3–4). In dorsal view, the prefrontal is anteroposteriorly elongated and lateromedially narrow, as in other basal sauropodomorphs (e.g. *Mussaurus*, *Massospondylus*, *Lufengosaurus*, *Adeopapposaurus*), but different from the transversely broader prefrontal of *Riojasaurus*, *Coloradisaurus*, *Plateosaurus*, and *Melanorosaurus.* The prefrontal contributes to the anterodorsal margin of the orbit (Figure 3B), as in most basal sauropodomorphs. Posteriorly the prefrontal fits into a triangular slot on the anterolateral region of the frontal. The posterior end of the prefrontal extends less than one-third of the anteroposterior dorsal margin of the orbit.

The left frontal is complete and better preserved than the right element (Figure 4). The frontal is longer than wide, transversely constricted at midsection, and reachs its maximum lateromedial width at its posterior end. The midline suture is straight at its anterior half, and interdigitated at its posterior half, similar to *Massospondylus*, *Melanorosaurus,* and *Adeopapposaurus*. The anterior region has a shallow and triangular depression for the nasal and a subtriangular slot for the reception of the prefrontal at its anterolateral region (Figure 4). The lateral margin of the frontal forms most of the dorsal rim of the orbit (Figure 3B, 4B), as in *Riojasaurus*, *Massospondylus*, *Pantydraco, Mussaurus, Yunnanosaurus,* and *Adeopapposaurus*, which differs from the small contribution to the orbital margin present in other taxa (e.g. *Plateosaurus*, *Coloradisaurus, Melanorosaurus, Lufengosaurus*). Posterolaterally, an elongated slot on the dorsal region of the frontal is overlapped by the anteromedial process of the postorbital (Figure 4B). Posteromedially, the frontal contacts the parietal but the location of the fronto-parietal suture cannot be precisely determined. The posterior edge of the frontal reaches the anterodorsal margin of the supratemporal fossa as in most basal sauropodomorphs.

The parietals are not fully preserved but it is clear that they are not medially fused (Figure 4). The anterolateral process of the parietal contacts the frontal and the postorbital and forms the medial half of the anterior wall of the antorbital fossa. The parietal-postorbital suture is located midway along the supratemporal fossa, as in *Massospondylus* and *Adeopapposaurus*. The posterolateral process tapers distally, and is lateroventrally overlapped by the medial process of the squamosal (Figure 4B). Similar to the condition of most basal sauropodomorphs, the posterolateral process forms an angle of approximately 45 degrees with the longitudinal axis of the skull (Figure 4). This process is ventrally deflected and forms the entire medial and posteromedial walls of the supratemporal fossa. Dorsally, the posterolateral process has a sharp dorsal margin. In posterior view, this process is strap-like and directed posterolaterally with a shallow concave surface.

Both postorbitals of *Leyesaurus* are completely preserved (Figure 3, 4) and these elements are proportionally wider and thicker than that in *Adeopapposaurus*. The postorbital of *Leyesaurus* has a long anteromedial and descending processes and a short posterior process. The main body of the postorbital is smooth and bears small foramina scattered on its lateral surface. The anteromedial process is the most robust of the three processes, with a tongue-shape widening distal end (Figure 4). The ventral portion of the anteromedial process contacts medially with the parietal, and both form the anteroventral wall of the supratemporal fossa. The dorsal portion of the anteromedial process fits into a slot on the posterolateral region of the frontal, being excluded from the dorsal margin of the orbit. The anterior edge of the main body of the postorbital and of the descending process forms the entire posterior orbital rim (Figure 3), as in *Riojasaurus*, *Coloradisaurus*, *Massospondylus*, *Lufengosaurus* and *Adeopapposaurus*. The descending process of the postorbital is thin and tapers distally. The shaft of the descending process is wider transversely than anteroposteriorly at its midlength (Figure 4). The latter condition is only shared with *Adeopapposaurus*, *Ignavusaurus*, *Anchisaurus*, and Neosauropoda. The posterior surface of the descending process has a deep groove that lodges the ascending process of the jugal. The posterior process of the postorbital is posteromedially directed, almost horizontal, and anteroposteriorly longer than deep (Figure 3). This process forms the lateral margin of the supratemporal fenestra and the dorsal margin of the infratemporal fenestra, similar to other basal sauropodomorphs.

The left jugal is complete but somewhat damaged and displaced from its natural position, whereas the right element is only partially exposed (Figure 3). The anterior suborbital process of the jugal is the most robust and forms the main body of the jugal. Approximately two thirds of its dorsal edge forms the ventral margin of the orbit. This process articulates the maxilla anteriorly and the lacrimal anteromedially. The minimum dorsoventral depth of the suborbital region of the jugal is slightly more than 20% of its anteroposterior length (measured from the anterior tip of the jugal to the anteroventral corner of the infratemporal fenestra, Figure 3), similar to other basal sauropodomorphs (*e.g. Coloradisaurus*, *Massospondylus*, *Lufengosaurus*, *Adeopapposaurus*). The anterior end of the jugal is pointed and reaches the posteroventral corner of the antorbital fossa. The posterior processes of the jugal are displaced from their original position and the angle between them cannot be calculated. The quadratojugal process is posteriorly directed, and extends approximately along two thirds of the ventral edge of the infratemporal fenestra, as in others basal sauropodomorphs (e.g. *Riojasaurus*, *Massospondylus*, *Yunnanosaurus*, and *Adeopapposaurus*).

The quadratojugals are partially exposed on both sides of the skull. Only the anterior process of the left element is preserved, whereas the right quadratojugal consists of an incomplete main body and anterior process (Figure 3). The main body of the quadratojugal is at the posteroventral corner of the infratemporal fenestra. The jugal and squamosal processes of the quadratojugal almost form a perpendicular angle in lateral view (Figure 3), as in the basal saurischian *Herrerasaurus* and some basal sauropodomorphs (e.g., *Eoraptor*, *Riojasaurus*, *Melanorosaurus*, *Unaysaurus*, *Adeopapposaurus*) and subadult specimens of *Massospondylus* and *Mussaurus*. The anterior process is slender and tapers distally, and extends along most of the ventral margin of the infratemporal fenestra.

Both quadrates are preserved but they are somewhat distorted and displaced from their natural position (Figure 3). The quadrate shaft is sinuous in lateral and posterior views. The dorsal head of the quadrate is suboval and forms an elongated condyle that abuts to the ventral region of the squamosal, at the level of the dorsal margin of the infratemporal fenestra. The shaft of the quadrate of *Leyesaurus* is dorsoventrally shorter and transversely narrower than in *Adeopapposaurus*, but the quadrate head of *Leyesaurus* is markedly reduced in size compared with *Adeopapposaurus*. The ventral third of the quadrate shaft is stout and subtriangular in cross-section. Its anterior surface is concave, whereas its posterior surface is convex and bears a dorsoventrally oriented ridge. The shaft expands ventrally to form the articular surface of the quadrate condyles. This articular area is subtriangular in ventral view and subdivided into a medial and a lateral condyle (Figure 3). The medial condyle is anteroposteriorly longer and projects more ventrally than the lateral one, as in other basal sauropodomorphs (e.g. *Pantydraco*, *Lufengosaurus*, *Massospondylus, Adeopapposaurus*). The quadrate foramen is a deep gap that opens on the anterior margin of the shaft, slightly posterior to the quadrate-quadratojugal suture. The two anterior processes of the quadrate are set at an angle of approximately 90 degrees to each other. The anteromedial process occupies more than 70% of the total length of the quadrate shaft, similar to *Plateosaurus, Lufengosaurus, Jingshanosaurus, Melanorosaurus, Unaysaurus,* and *Adeopapposaurus*; but differs from other sauropodomorphs in which this process does not exceed 70% of the total quadrate height (e.g. *Saturnalia*, *Coloradisaurus, Massospondylus, Pantydraco*, *Mussaurus*).

The squamosal is a tetraradiate element that forms the posterolateral corner of the supratemporal fenestra and the posterodorsal corner of the infratemporal fenestra (Figure 4). The complete right squamosal of *Leyesaurus* is disarticulated from the skull, whereas the left element has preserved its descending and anteromedial processes in articulation with the quadrate and the parietal, respectively. The anterolateral process of the squamosal is short and broad, and V-shaped in lateral view. The descending process is the longest, anteroventrally directed, and ventrally tapered. Laterally, the descending process diverges from the anterolateral process at an angle of approximately 45 degrees. The posteroventral process is short, laminar, and tapers distally. In lateral view, the posteroventral and the descending process of the squamosal form an angle of approximately 90 degrees. The anteromedial process is laminar (Figure 4A) and has a medial sulcus along its contact with the parietal.

#### Palate

Most of the palatal elements are damaged, incompletely preserved, and overlap each other, precluding the observation of anatomical details.

The ventral side of both ectopterygoids is exposed in ventral view, but both are displaced from their natural position. The main body of the ectopterygoid has a typical flat triangular shape with a slender and strongly recurved anterolateral process. Its ventral surface is smooth and bears a shallow depression on its posteromedial area. The ectopterygoid of *Leyesaurus* lacks the pneumatic fossa present in *Pantydraco*.

Both pterygoids are partially exposed in ventral view. The main body of the pterygoid is anteroposteriorly longer than transversely wide, with an irregular ventral surface. Its posteromedial process is narrow and wraps around the basipterygoid process, as in other basal sauropodomorphs (*e.g. Plateosaurus*, *Adeopapposaurus*), and differs from the broad curved process of *Riojasaurus* and *Melanorosaurus*. The transverse process is slender and extends laterally from the main body of the pterygoid. This process curves anterolaterally and contacts the medial side of the mandible. Other details of the pterygoid cannot be observed.

#### Braincase

The occipital region has been strongly eroded and is missing most of its elements. Only the basioccipital and the posterior half of the basisphenoid-parasphenoid complex have been preserved.

The dorsal surface of the basioccipital is anteroposteriorly elongated (Figure 4B) and forms most of the floor of the endocranial cavity. The anterior half of the dorsal surface is wider than the posterior one and has a longitudinal medial ridge. The anterior edge is transversely straight and contacts the posterior edge of the basisphenoid. The posterior dorsal half of the basioccipital has a longitudinal median sulcus flanked by two anteroposteriorly elongated lateral facets (Figure 4B) where the exoccipitals articulate. In lateral view, the main body of the basioccipital is anteroventrally curved, with its anteroventral margin that extends well below the ventral margin of the occipital condyle. This low region is laterally expanded and forms the basioccipital component of the basal tubera. The basioccipital condyle has a suboval shape with a convex posterior and ventral surface. In posterior view, the basioccipital condyle of *Leyesaurus* is transversely wider but dorsoventrally shorter than that in *Adeopapposaurus*.

The dorsal surface of the basisphenoid forms the anterior region of the floor of the braincase. This surface is concave and strongly rugose, and contacts the anterior edge of the basioccipital posteriorly. The anterior half of the basisphenoid of *Leyesaurus* is transversely narrower than the posterior half. The basipterygoid processes are laterally compressed and extend ventrolaterally as well as anteriorly. These processes are set at an approximately perpendicular angle. The basipterygoid processes of *Leyesaurus* are anteroposteriorly and transversely thinner than those of *Adeopapposaurus,* and lack the expanded distal end present in that taxon. Moreover, in ventral view the basispterygoid processes of *Leyesaurus* extend laterally to approximately the level of the basal tuberae, whereas in *Adeopapposaurus* these processes extend laterally well beyond the level of the basal tuberae. The basisphenoid component of the basal tuberae is directed laterally and dorsally expanded. Overall, the basioccipital and the basisphenoid component of the basal tuberae of *Leyesaurus* are lateromedially wider than those in *Adeopapposaurus*. The parasphenoid cannot be observed. The basal tuberae, the bases of the basipterygoid processes, and the parasphenoid rostrum are approximately aligned in lateral view, as in some basal sauropodomorphs (e.g. *Efraasia*, *Thecodontosaurus* (YPM 2192), *Massospondylus*, *Anchisaurus*, *Adeopapposaurus*).

#### Mandible

Most of the right mandibular ramus is preserved and exposed in lateral view, whereas only the posterior half of the left ramus has been preserved (Figure 2–4). As preserved, the mandible is shorter than the skull length, although the angular, surangular, and articular are incomplete and severely distorted (see Table S1 for measurements).

The dentary is the largest bone of the lower jaw (Figure 3). Its anteroposterior length is five times its maximum depth and its dorsal and ventral borders are parallel to each other. In lateral view, the anterior end of the dentary has a rugose surface with several foramina scattered around the symphyseal region (Figure 3B), resembling the condition of *Adeopapposaurus*. A row of foramina extends parallel to and just below the alveolar margin of the dentary (Figure 3B). These foramina open anterodorsally, with the exception of the posteriormost foramen that opens posteriorly. This foramen is larger, and spaced further from the others. The first dentary alveolus is inset a short distance from the symphysis (less than the width of an alveolus) as in *Panphagia*, *Pantydraco*, and *Anchisaurus*. The dentigerous portion occupies almost the entire length of the preserved dorsal margin of the dentary.

The angular, surangular, and articular are incomplete, partially exposed, and severely distorted (Figure 3), hence they do not offer many details for description. Overall, the coronoid eminence of *Leyesaurus* is strongly arched, and the convexity of the dorsal margin is more pronounced than that of *Adeopapposaurus*. Moreover, the coronoid eminence of *Leyesaurus* tapers posteriorly to form the retroarticular process that exhibits an abrupt concave curvature in lateral view (Figure 3B), whereas in *Adeopapposaurus* this curvature is much less pronounced. The splenial and intercoronoid cannot be identified because they are obscured by the matrix and other bones on both sides.

#### Dentition

**Figure 5:**
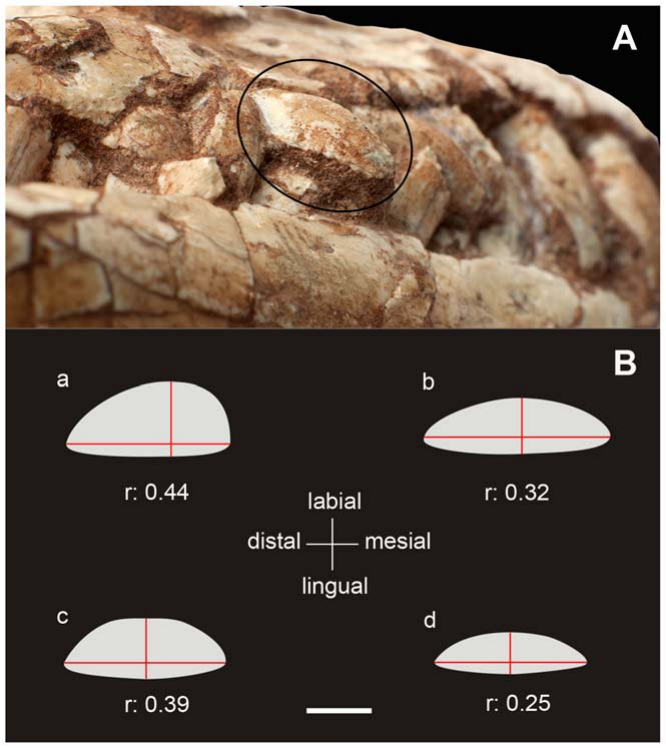
Maxillary teeth of basal sauropodomorphs. A, photograph of the teeth of *Leyesaurus marayensis* (PVSJ 706), the circle shows fifth maxillary tooth in distal view; B, Cross-sections at mid-height of an anterior maxillary tooth of *Leyesaurus* PVSJ 706 (a), *Adeopapposaurus* PVSJ 568 (b), *Massospondylus* SAM-PK-K1314 (c), and *Riojasaurus* PVSJ 849 (d). Red lines represent the maximum mesiodistal length and labiolingual width at mid-height of the tooth. *Abbreviations*: *r*, ratio between mesiodistal length and labiolingual width. Scale bar equals 2 mm.

*Leyesaurus* has a moderate degree of heterodonty (Figure 3). All preserved crowns are leaf-shaped and longer apicobasally than they are wide mesiodistally. The teeth are constricted between the crown and the root and the maximum mesiodistal expansion of the crown is located on the proximal third. The upper tooth crowns have a markedly convex labial side (Figure 5A) whereas dentary tooth crowns are compressed labiolingually with slightly convex labial sides.

*Leyesaurus* has four premaxillary teeth, which are the longest elements of the dentition (Figure 3B). The mesial and distal margins of the premaxillary teeth are asymmetric and lack serrations. The apices of the premaxillary teeth are pointed and lingually inclined.

The maxillary teeth are shorter than those of the premaxilla (Figure 3). Maxillary and dentary crowns are almost symmetrical in labial view, and bear blunt denticles along the apical two-thirds of the mesial and distal margins. The denticles are oriented at approximately 45 degrees from the edge. Unlike *Adeopapposaurus*, all denticles of *Leyesaurus* form an angle of 45 degrees with the basal-apical axis of each crown, whereas some maxillary teeth of *Adeopapposaurus* have parallel serrations.

Labial surfaces of the maxillary and dentary crowns of *Leyesaurus* are smooth and lack the moderate enamel wrinkling (Figure 3, 5A) present in *Anchisaurus* [42], or the more developed wrinkling of sauropod taxa [35]. The labial surfaces of the anterior maxillary teeth are strongly convex, whereas the lingual surfaces are almost flat and D-shaped in cross-section (Figure 5). This convex labial surface of the anterior maxillary teeth has the labial highest point mesially displaced, and gives an irregular D-shaped cross-section of the tooth (Figure 5B). The noticeably bulging labial side of the maxillary teeth of *Leyesaurus* is more pronounced than that of other basal sauropodomorphs (e.g., *Massospondylus, Mussaurus, Anchisaurus, Plateosaurus*), and represents a distinctive feature of the upper teeth of *Leyesaurus* (Figure 5) (See Discussion).

In many respects, the dentary teeth exhibit a similar morphology as the maxillary teeth, although the dentary teeth are more flattened labiolingually. Moreover, the dentary teeth have their maximum mesiodistal width closer to the base than the maxillary teeth. The anterior dentary teeth are slightly longer than the posterior dentary teeth, similar to most basal sauropodomorphs. The labial surface of the crowns is slightly convex, while the lingual surface is slightly convex to flat. Dentary teeth are slightly rotated giving an imbricated arrangement, similar to other basal sauropodomorphs such as *Massospondylus*, *Yunnanosaurus*, *Thecodontosaurus* (BRSMG C4529), *Lufengosaurus*, *Adeopapposaurus*, premaxillary teeth of *Mussaurus*, as well as some teeth of *Plateosaurus* and *Ignavusaurus*.

#### Ceratobranchial

A pair of ceratobranchials is joined to the palatal elements, and they extend anteroposteriorly. The ceratobranchials are thin, elongate and gently curved along their length. They are subcircular in cross section and are almost constant in diameter, with blunt anterior and posterior ends.

### Axial skeleton

#### Atlas–axis complex

**Figure 6:**
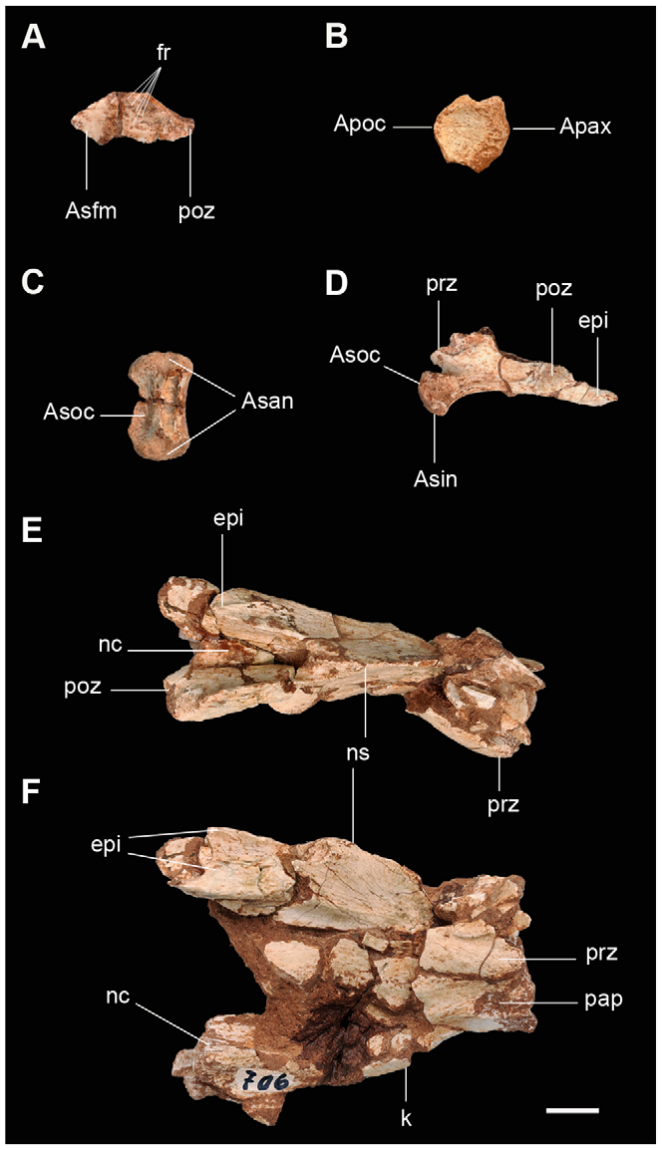
Atlas-axis complex of the new basal sauropodomorph *Leyesaurus marayensis* (PVSJ 706). A, right proatlas in medial view; B, odontoid in dorsal view; C, intercentrum in dorsal view; D, right atlantal neural arch in medial view; E–F, axis in dorsal (E) and lateral (F) view. *Abbreviations*: *Apax*, articular projection with the axis; *Apoc*, articular projection with the occipital; *Asan*, articular surface with the atlantal neural arch; *Asfm*, articular surface with the lateral region of the foramen magnum; *Asin*, articular surface with the intercentrum; *Asoc*, articular surface with the occipital; *epi*, epipophysis; *fr*, foramen; *k*, keel; *nc*, neural canal; *ns*, neural spine; *pap*, parapophysis; *poz*, postzygapophysis; *prz*, prezygapophysis. Scale bar equals 1 cm.

The right proatlas lacks its posterior end (Figure 6A) and the left proatlas lacks its posterior half. In lateral view, the proatlas is subtriangular and transversely flattened (see Table S1 for measurements), unlike the subrhomboidal proatlas of *Adeopapposaurus*. The anterior end has a rectangular surface that faces anterolaterally and articulates with the dorsolateral region of the foramen magnum. The anterodorsal border of the proatlas is rounded, anteroposteriorly directed, and contacts the other proatlas to form the roof of the neural canal (Figure 6A). The dorsal end of the proatlas bears a lateral bulbous projection. The medial surface is slightly concave whereas the lateral side has an undulated surface, and several foramina are present on both sides. The laminar posterior end, or postzygapophysis, presents a smooth medial surface for the articulation with the prezygapophysis of the atlantal neural arch (Figure 6A).

All elements of the atlas are disarticulated, and consist of the odontoid, intercentrum, and both incomplete neural arches (Figure 6B–D; see Table S1 for measurements). In dorsal view the odontoid is subcircular with a concave surface (Figure 6B). Its anterior margin bears a pointed projection that fits on the occipital condyle. Ventrally, the odontoid has a transversely directed deep groove covered by several foramina, which articulates with the posterodorsal margin of the atlantal intercentrum. The posterior surface bears a rounded projection on its dorsal third that fits on the anterior articular surface of the axial centrum (Figure 6B).

The intercentrum is subrectangular U-shaped in anterior and posterior views and subtriangular in lateral view. The dorsal surface of the intercentrum bears two transversely oriented concavities for the articulation of the occipital condyle (anteriorly) and the odontoid (posteriorly) (Figure 6C). The posterior surface of the intercentrum is convex with a concave dorsal margin and a straight ventral margin. The ventral surface is rugose and slightly concave. The circular articular surfaces for the atlantal ribs are located on the lateral side of the odontoid, and face dorsolaterally.

The right atlantal neural arch is almost complete whereas only the anterior half of the left neural arch has been preserved (Figure 6D). The atlantal neural arch is anteroposteriorly elongated, approximately three times longer than high (measured from the anterior end of the pedicle to the posterior end of the postzygapophysis), as in *Adeopapposaurus* (see Table S1 for measurements). The medial surface is smooth and anteroposteriorly concave. The anteroventral corner bears a subtriangular pedicle that articulates ventrally with the laterodorsal depression of the intercentrum and anteriorly with the occipital region. Anterodorsally, the atlantal neural arch has an anteromedially directed bilobated lamina that forms the lateral wall of the neural canal (Figure 6D). The anterolateral surface of this lamina forms the prezygapophysis that articulates with the proatlas. The postzygapophysis is tongue-shaped with a pointed posterior end (Figure 6D). The epipophysis is medially curved, projects posteriorly, and overhangs the posterior end of the postzygapophysis (Figure 6D).

The length of the axial centrum is more than four times longer than high and anteroposteriorly shorter than any of the postaxial cervical (Figure 2, 6E–F; see Table S1 for measurements). The shape of the axial centrum of *Leyesaurus* differs from other basal sauropodomorphs where the length:height ratio approximately ranges between 2 and 3.5 (*Yunannosaurus*, 2.1; *Melanorosaurus*, 2.5; *Unaysaurus*, 2.0; *Riojasaurus*, 3.3; and *Adeopapposaurus*, 3.5).The ventral surface of the axis bears a ventral keel that extends along the entire length of the centrum (Figure 6F), similar to *Unaysaurus* and *Adeopapposaurus*. Other sauropodomorphs lack a ventral keel on the axis (e.g. *Riojasaurus* and *Thecodontosaurus* (BMNH P24)). The anterior articular surface is markedly rugose and bears a circular concavity at its dorsal third, which lodges the posterior projection of the atlantal odontoid. The lateral surface of the centrum is not fully preserved, so that the diapophyses cannot be identified, whereas the parapophyses are small and shallow depressions located on the anteroventral region of the centrum (Figure 6F). Most of the neural arch of the axis is broken (Figure 6E–F). The prezygapophyses are small and smooth surfaces placed at anterior and of the neural arch. The postzygapophyses are medially connected by a thin lamina that forms the roof of the posterior exit of the neural canal. The articular surfaces of the postzygapophyses are elongated and face ventrolaterally. The posterior end of the centrum is poorly preserved and it is not possible to determine whether the postzygapophyses extend beyond the posterior margin of the centrum (Figure 6F). The neural spine only preserves its posterior half, which is low, laminar, and thickens at its posterior end. Posteriorly, the neural spine is divided into two spinopostzygapophyseal laminae that project ventrolaterally and merge distally with the epipophyses. The epipophyses are robust, have a sharp dorsal margin, and extend along two-thirds of the total length of the dorsal surface of the postzygapophyses (Figure 6E–F).

#### Cervical vertebrae

**Figure 7:**
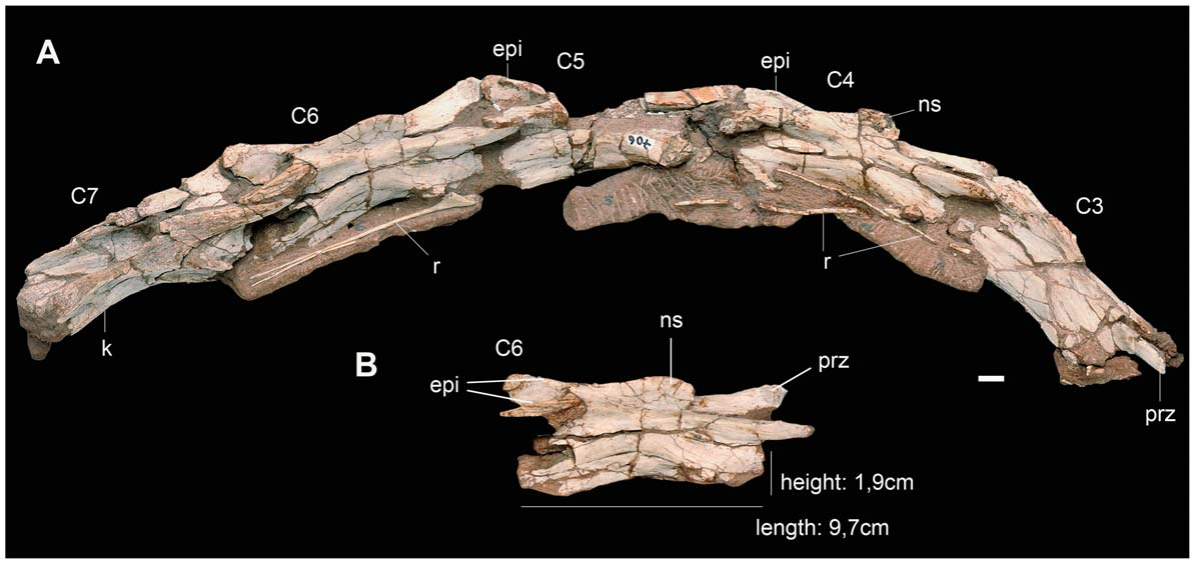
Cervical vertebrae of the new basal sauropodomorph *Leyesaurus marayensis* (PVSJ 706). A, neck vertebrae from C3–C7; B, sixth cervical vertebra in lateral view. *Abbreviations*: *C3*–*7*, cervical vertebrae; *epi*, epipophysis; *k*, keel; *ns*, neural spine; *prz*, prezygapophysis; *r*, ribs. Scale bar equals 1 cm.

The preserved postaxial cervical vertebrae are articulated from the third to the seventh vertebra (Figure 2, 7A). All preserved postaxial cervical vertebrae are low, elongate and lateromedially compressed at their midlength (Figure 7A). All postaxial centra are longer than the axial centrum and their length increases slightly posteriorly (see Table S1 for measurements). The centra are approximately 1.25 times higher than wide, lateromedially constricted at their midlength, and ventrally keeled (Figure 7A). The third cervical centrum is 30% longer than the axial centrum similar to *Herrerasaurus* and other basal sauropodomorphs (such as *Riojasaurus, Plateosaurus* and *Yunnanosaurus*), but different from the much more elongated C3 of *Coloradisaurus* and *Adeopapposaurus* (which is twice as long as the axis). The anteroposterior length of the anterior cervical centra is more than 4 times its dorsoventral height, whereas the sixth vertebra has the greatest elongation, with a centrum 5.12 times longer than tall (Figure 7B; see Table S1 for measurements). The sixth cervical vertebra of *Leyesaurus* is proportionately more elongated than other non-eusauropod sauropodomorphs, in which the height/length ratio of the cervical vertebrae varies between 3 and 4 (see Discussion). The ventral surface of the centra is concave. Cervical vertebrae 3 to 6 have a ventral keel on their anterior half (as in *Thecodontosaurus* (YPM 2192) and *Lamplughsaura*), whereas the centrum of cervical 7 has a keel that extends along its entire length, similar to *Yunnanosaurus*. This condition is different from that of some basal sauropodomorphs (e.g. *Massospondylus*, *Panphagia*, *Eoraptor, Adeopapposaurus*) and the basal saurischians *Herrerasaurus*, in which all the anterior cervical centra are keeled along their entire ventral surface. Other basal sauropodomorphs, instead, lack a ventral keel in the anterior and middle cervical centra (e.g. *Riojasaurus*, *Pantydraco*, *Plateosaurus, Lessemsaurus*). The parapophyses are located at the anterodorsal region of the lateral surface of the centrum, and the diapophyses are small protuberances near the anterior edge of the centra. All zygapophyses extend parallel to the anteroposterior axis of the centrum and their length is greater than the maximum centrum length (Figure 7A). The epipophyses extend along two thirds of the total length of the postzygapophyses (Figure 7B), as in *Massospondylus* (BP/1/4934). The latter condition is different from that of other basal sauropodomorphs, in which epipophyseal length exceeds two-thirds of the length of the postzygapophyses or in which the epipophyses reach the posterior margin of the postzygapophyses (see Discussion). The neural spine is low and transversely compressed. In lateral view, the dorsal border of the neural spines is anteriorly convex and slightly concave posteriorly (Figure 7), similar to *Massospondylus* (Cooper, 1981: Fig. 5), but unlike other basal sauropodomorphs in which the neural spine has straight or convex dorsal border (see Discussion).

All cervical ribs of *Leyesaurus* are oriented parallel to the longitudinal axis of their respective vertebrae (Figure 7A). The shaft of the cervical ribs is slender, elongated, and circular in cross-section. The capitular and tubercular processes of the anterior cervical ribs are poorly developed, and the tubercular process is smaller than the capitular process. The posterior cervical ribs are slightly more robust and their capitular and tubercular processes are more developed than in the anterior cervical ribs.

#### Caudal vertebrae

**Figure 8:**
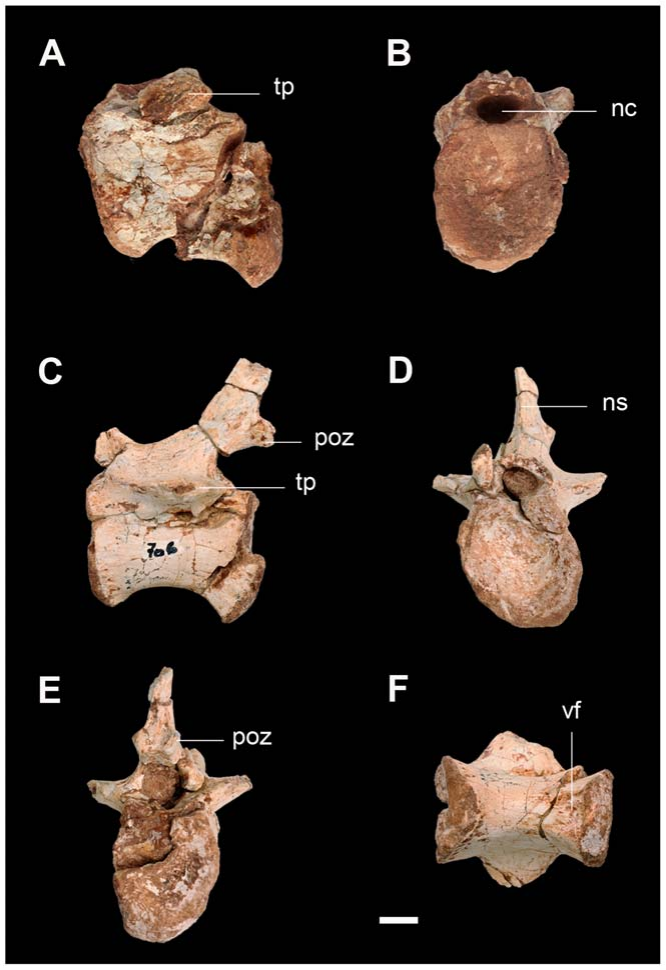
Caudal vertebrae of the new basal sauropodomorph *Leyesaurus marayensis* (PVSJ 706). A–B, anterior caudal vertebra in lateral (A) and anterior (B) view; C–F, middle caudal vertebra in lateral (C), anterior (D), posterior (E), and ventral (F) view. *Abbreviations*: *nc*, neural canal; *ns*, neural spine; *poz*, postzygapophysis; *tp*, transverse process; *vf*, ventral furrow. Scale bar equals 1 cm.

Only two non-consecutive caudal vertebrae are preserved in *Leyesaurus* (Figure 2). The larger one belongs to the anterior region whereas the other probably belongs to the middle section (Figure 8; see Table S1 for measurements). The two caudal vertebrae have amphicoelous centra, are slightly elongated and are slightly compressed lateromedially with pronounced concave lateral and ventral sides (Figure 8A–F). The length of the two caudal centra is greater than their height at its anterior articular face (Figure 8A, C). The anteriormost caudal is more robust and has a length/height ratio of 1.2, whereas the other centrum has a ratio of 1.4. Each caudal centrum has ventrally expanded ventral edges of both articular surfaces, with the posterior ventral rim more developed than the anterior one (Figure 8A, C, E). The expanded border of the posterior articular surface has two flat articular facets for the haemal arch. In ventral view, both centra have a furrow that extends along the posterior third of their ventral surfaces that occupies the entire transverse width of the centrum (Figure 8F). Both caudal vertebrae have an incomplete neural arch, lacking prezygapophyses, the distal region of the transverse processes and part of the neural spine (Figure 8). The neural arch is saddle-shaped, as in most basal sauropodomorphs (Figure 8C). The transverse processes are completely fused to the centrum, dorsolaterally oriented, and arise at the anteroposterior midpoint of the neural arch. The neural spine is posterodorsally oriented and the length of its base is less than half the length of the neural arch (Figure 8C). The postzygapophyses are reduced in size and positioned near the base of neural spine, and they protrude somewhat beyond the posterior margin of the centrum (Figure 8C).

Only two isolated chevrons are preserved but they lack their distal ends. As in other basal sauropodomorphs, the shafts are rod-like with a forked proximal end, which produces the characteristic “Y” shape. In one of the chevrons the branches of the proximal end are fused and form an oval concave facet for articulation with the centrum, and likely indicate that it might belong to the anterior-mid caudal region. Its shape resembles to that of Adeopapposaurus and Pantydraco. The other chevron is more robust and its arms are proximally separated, suggesting that it belongs to a mid-posterior position in the caudal series. The proximal half of the posterior face of the larger chevron has a deep groove running between both arms which form the oval foramen for the caudal blood vessels.

#### Pectoral girdle and forelimb

**Figure 9:**
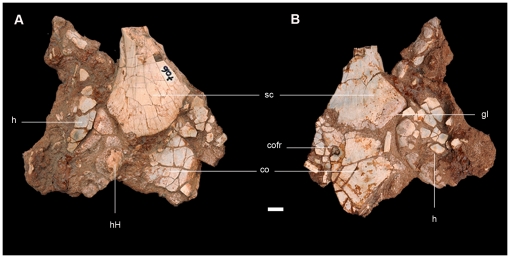
Preserved elements of the pectoral girdle of the new basal sauropodomorph *Leyesaurus marayensis* (PVSJ 706). A–B, left scapula, coracoid, and humerus in medial (A) and lateral (B) view. *Abbreviations*: *co*, coracoid; *cofr*, coracoid foramen; *gl*, glenoid surface; *h*, humerus; *hH*, humeral head; *sc*, scapula. Scale bar equals 1 cm.

The preserved elements of the pectoral girdle and forelimb are the proximal region of the left scapula, a damaged and dorsoventrally compressed left coracoid, and a poorly preserved proximal fragment of the left humerus (Figure 2, 9; see Table S1 for measurements). All bones are only partially preserved, cracked, and distorted so that anatomical details cannot be interpreted with certainty in these elements.

#### Pelvic girdle

**Figure 10:**
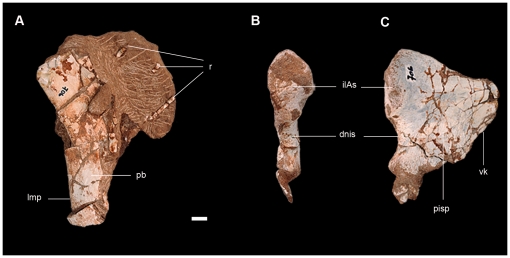
Preserved elements of the pelvic girdle of the new basal sauropodomorph *Leyesaurus marayensis* (PVSJ 706). A, right pubic apron in dorsal view; B–C, proximal portion of the left ischium in proximal (B) and lateral (C) view. *Abbreviations*: *dnis*, dorsal notch of the ischial symphysis; *ilAs*, iliac articular surface of the ischium; *lmp*, lateral margin of the pubic apron; *pb*, pubic apron; *pisp*, proximal ischial portion; *r*, ribs; *vk*, ventral keel of the ischial shaft. Scale bar equals 1 cm.

Only the right pubic apron and the proximal portion of both ischia have been preserved in the type specimen of *Leyesaurus* (Figure 2, 10; see Table S1 for measurements).

The pubic apron has several transverse fractures and lacks its proximal and distal ends (Figure 10A). The shaft is oval in cross-section with a concave and rounded lateral margin, and a sharp medial edge, similar to most basal sauropodomorphs. The left ischium is partially represented by its proximal portion, whereas the right element consists only of the articular surfaces with the ilium and pubis (Figure 10B,C). The proximal plate of the left element is thin with a slightly convex lateral surface and a concave medial surface. The dorsolateral margin is lateromedially thicker and rounded whereas the ventromedial margin is laminar and ends in a sharp edge. A longitudinal sulcus is present at the dorsolateral margin of the proximal region of the left ischium. The articular surface with the ilium is suboval in cross-section and has an irregular surface. The articular surface for the pubis is not completely preserved but it is smaller than the articular surface for the ilium (Figure 10B).

#### Hindlimb

The hindlimb of *Leyesaurus* consists of an incomplete left pes, including the distal tarsals III and IV, proximal half of metatarsal III, complete metatarsals IV and V, first phalanx of digit I, second phalanx of digit II, and second phalanx of digit IV (Figure 2, 11–12; see Table S1 for measurements).

**Figure 11:**
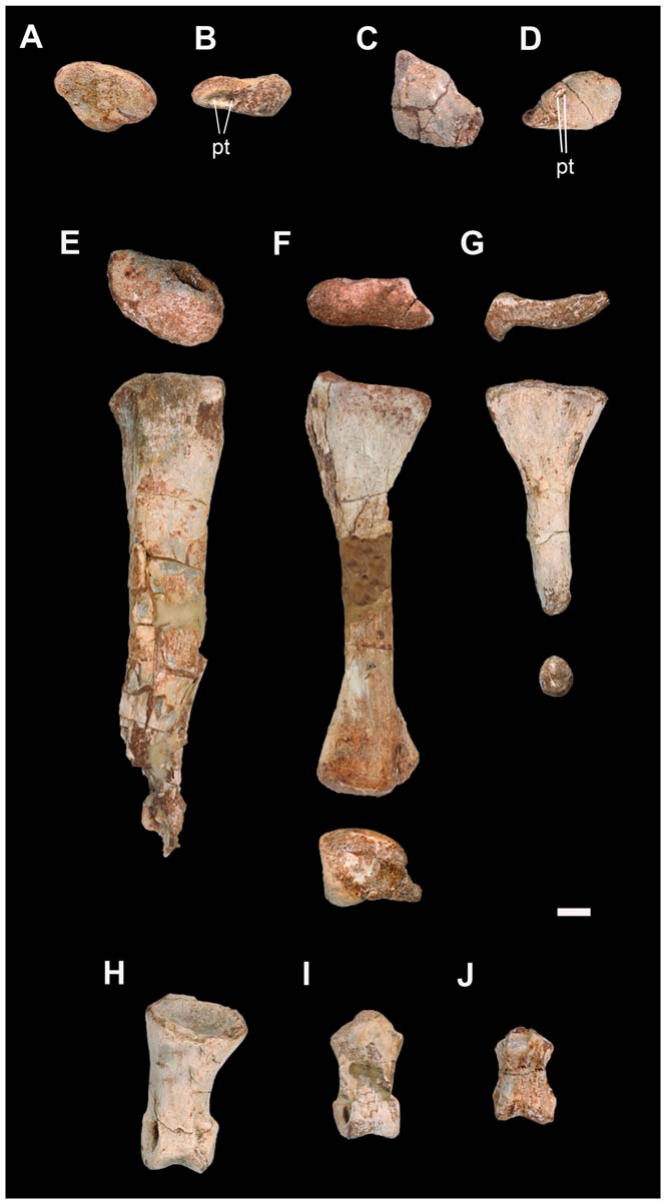
Foot elements of the new basal sauropodomorph *Leyesaurus marayensis* (PVSJ 706). A–B, left distal tarsal III in dorsal (A) and posterior (B) view; C–D, left distal tarsal IV in dorsal (C) and posterior (D) view; E–G, left metatarsal III (E), IV (F), and V (G) in proximal, dorsal and distal view; H–J, left pedal phalanges: first phalanx of digit I (H), second phalanx of digit II (I), and second phalanx of digit IV (J) in dorsal view. *Abbreviations*: *pt*, ligament pit. Scale bar equals 1 cm.

**Figure 12:**
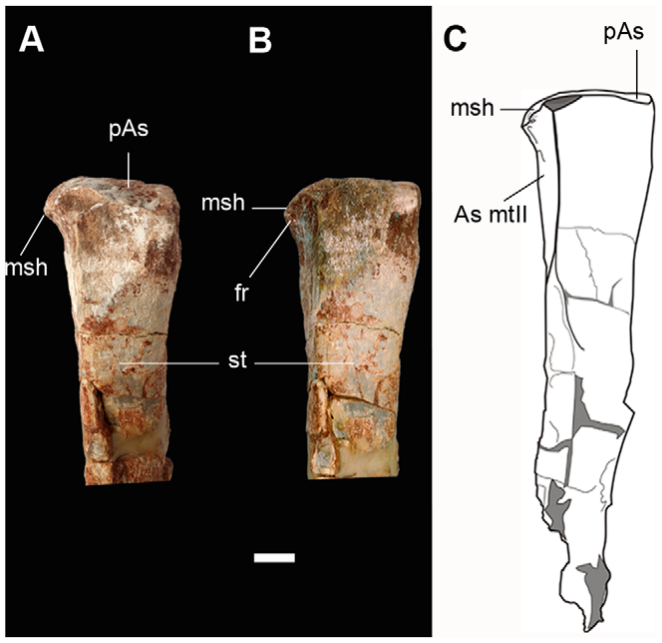
Metatarsal III of the new basal sauropodomorph *Leyesaurus marayensis* (PVSJ 706). A–B, photograph of the proximal half of metatarsal III in dorsal (A) and dorsomedial (B) view; C, interpretative drawing showing the medial shelf-like deflection of metatarlas III. *Abbreviations*: *As mtII*, articular surface for the metatarsal II; *fr*, foramen; *msh*, medial shelf of the metatarsal III; *pAs*, proximal articular surface; *st*, shaft of the metatarsal III. Scale bar equals 1 cm.

Distal tarsal III is proximodistally flat, has a subtriangular shape and its major axis is oriented anterolaterally (Figure 11A–B). The anterolateral region is proximodistally thin and increases in thickness towards its posteromedial region, where the distal tarsal III reaches its deepest portion (Figure 11B). The posterolateral surface has two ligament pits (Figure 11B) and the posteromedial surface bears a large pit that is internally divided. Distal tarsal IV is hemicone shaped and is larger than distal tarsal III (Figure 11C–D). It has a subtriangular shaped proximal and distal surface and is subrhomboidal in posterior view. The major axis of distal tarsal III is anterolaterally oriented, and its posteromedial region is the proximodistally deepest part of the bone (Figure 11D), similar to *Adeopapposaurus*. On the anterior border and posterolateral surface, distal tarsal IV has two deep pits located within an elongated furrow.

Metatarsal III lacks its distal end (Figure 11E, 12). The proximal articular surface of metatarsal III is subtriangular, similar to *Saturnalia, Thecodontosaurus* (BRSMG Ca7451a), *Massospondylus, Lufengosaurus, Lessemsaurus,* and *Adeopapposaurus*. In medial view, metatarsal III has a smooth triangular surface for the articulation of metatarsal II. The medial margin of the proximal articular surface of metatarsal III has a distinct medial projection (Figure 11E, 12). This medial process is hook-shaped (Figure 12A), and may overlap the lateral margin of the proximal articular surface of metatarsal II. On this medial surface, ventrally to the hook-shaped process, metatarsal III has a deep elongated foramen (Figure 12). This distinctive feature of the proximal end of the metatarsal III is absent in all other basal sauropodomorphs (see Discussion). The preserved fragment of the proximal half of the shaft of metatarsal III is dorsoventrally flattened and has an elliptical cross-section (Figure 11E). Metatarsal IV is slender and less robust than metatarsal III (Figure 11F). As in most sauropodomorphs, metatarsal IV is broader at its proximal end than at its distal end. The proximal articular surface is dorsoventrally flat, has a subtriangular outline, and is almost three times as wide as high (Figure 11F), similar to other basal sauropodomorphs (e.g. *Massospondylus, Pantydraco, Adeopapposaurus*). The proximal end of the metatarsal IV has a large concavity on its ventrolateral region for contact with metatarsal V. The shaft of metatarsal IV is dorsoventrally compressed and has an elliptical cross-section. The transverse width of the distal end of metatarsal IV is larger than its dorsoventral height, and the lateral condyle extends ventrolaterally as a well-developed flange (Figure 11F). The dorsal fossa for the extensor ligament is absent in metatarsal IV. The distal articular surface of metatarsal IV lacks the intercondylar groove, similar to *Riojasaurus* and *Adeopapposaurus*. As in all basal sauropodomorphs, metatarsal V is dorsoplantarly flat and funnel-shaped (Figure 11G). The proximal articular surface of metatarsal V is dorsoventrally compressed and lateromedially broad, with a lateral dorsoventral expansion and a sinuous medial half (Figure 11G). The transverse width of its proximal end is slightly more than 50% its proximodistal length. Metatarsal V tapers distally along its length and has a rounded distal end (Figure 11G).

The pedal phalanges of *Leyesaurus* have a similar general morphology to those of other basal sauropodomorphs (e.g. *Saturnalia*, *Riojasaurus*, *Massospondylus*, *Pantydraco*, *Adeopapposaurus*, *Seitaad*), and are proximodistally longer than transversely wide, with a lateromedial constriction at their midshaft (Figure 11H–J). The distal condyles of the three preserved phalanges are broader at their ventral end than at their dorsal end, and have a well-developed intercondylar groove. The proximal end of the first phalanx of digit I and the second phalanx of digit II is wider than the distal end, whereas the proximal end of the second phalanx of digit IV is lateromedially narrower than the distal end. Their distal ends have lateral ligament pits that are deeper and lateroventraly more expanded than the medial ones (Figure 11H–J).

## Discussion

*Leyesaurus marayensis* has several features that distinguish it from all other known basal sauropodomorphs:

1- *Acute angle formed by the ascending process of the maxilla with the alveolar margin*. In basal sauropodomorphs the ascending process of the maxilla diverges from the posterior process at an angle that varying from approximately 60 to 90 degrees. In most basal sauropodomorphs the angle is almost perpendicular (e.g. *Coloradisaurus*, *Massospondylus*, *Melanorosaurus*, *Lufengosaurus*, *Efraasia*, *Adeopapposaurus*, posthatchling specimens of *Mussaurus* [19]; in others the angle varies between 65 to 80 degrees (e.g. *Riojasaurus*, *Plateosaurus*, *Jingshanosaurus*, *Unaysaurus*, subadult specimens of *Mussaurus)*; whereas in the Asiatic taxa *Yimenosaurus* and *Yunnanosaurus* the angle is approximately 60 degrees. In *Leyesaurus* the angle between the ascending process and posterior process of the maxilla is 50 degrees, the lowest value currently known among all non-eusauropod sauropodomorphs (Figure 3).

2- *Straight ascending process of the maxilla with a longitudinal ridge on its lateral surface*. As noted by Pol and Powell [19], the ascending process of the maxilla of some basal sauropodomorphs is posteriorly deflected at the location where the nasal overlaps the maxilla. Thus, the ascending process of some basal sauropodomorphs (e.g. *Melanorosaurus*, *Mussaurus*) has a distinct deflection along its dorsal-most region. In other forms (e.g. *Plateosaurus*, *Lufengosaurus*, *Efraasia*, *Anchisaurus*, *Coloradisaurus*, *Riojasaurus*), the ascending process is deflected at the dorsoventral midpoint of the antorbital fenestra, whereas in *Yimenosaurus* this process is slightly deflected at its ventral-most region. Unlike all the above-mentioned taxa, the ascending process of *Leyesaurus* is not deflected along its entire length (Figure 3). This condition is only shared with *Adeopapposaurus* and *Massospondylus* (SAM PK K1314; A. Yates, pers. com.). On the other hand, the lateral surface of the ascending process of *Leyesaurus* bears a rounded ridge throughout its entire dorsoventral length, a feature that is absent in all other basal sauropodomorphs. This ridge arises from the base of the lateral surface of the ascending process and continues tapering upwards. Anteriorly, this ridge forms a platform of the ascending process of the maxilla, which was probably overlapped by the nasal. Anterior and posterior to this ridge, the ascending process is transversely thin and sharp (Figure 3).

The presence of both aforementioned features—lack of deflection of the ascending process with a ridge running along its entire lateral surface—gives a unique combination of characters that is only present in *Leyesaurus marayensis*.

3- *Markedly bulging labial side of the maxillary teeth.* The teeth of basal sauropodomorphs are labiolingually compressed, with the labial surface slightly more mesiodistally convex than the lingual surface [2]. The maxillary teeth of *Leyesaurus* have a nearly flat lingual surface but are strongly convex on their labial side (Figure 5A), with a D-shaped cross-section that is more pronounced than in any other basal sauropodomorph (Figure 5B). Thereby, in *Leyesaurus* the labiolingual width at the midheight of the fourth maxillary tooth is 0.44 times its mesiodistal length. This ratio is smaller in other basal sauropodomorphs, such as *Adeopapposaurus* (0.32), *Massospondylus* (SAM PK K1314) (0.39), and *Riojasaurus* (PVSJ849) (0.25) (Figure 5B).

4- *Greatly elongated cervical vertebra —sixth cervical centrum with length/height ratio: 5.1.* The presence of elongated cervical vertebrae —at least twice as long as high— form a long neck that is a synapomorphic character of Sauropodomorpha [2], [43]. The anteroposterior length of the anterior cervical centra of *Leyesaurus* is more than 4 times the dorsoventral height of their anterior faces, whereas the longest cervical centrum (C6) is 5.1 times longer than its height (Figure 7B). The largest cervical length/height ratio among noneusauropod sauropodomorphs is slightly more than 4, as in *Massospondylus* (BP/1/4934), *Coloradisaurus*, *Lufengosaurus*, and *Adeopapposaurus*. In other basal sauropodomorphs (e.g. *Plateosaurus* [16], *Saturnalia, Riojasaurus*, *Lessemsaurus*, *Melanorosaurus, Eoraptor*, *Panphagia*), and the basal saurischians *Herrerasaurus* and *Sanjuansaurus*, the ratio is less than 3. Moreover, the abrupt elongation on the anterior cervical vertebrae of *Leyesaurus* (with a length/height ratio of more than 5) is unique among basal sauropodomorphs but resembles the extremely long cervical vertebrae of derived eusauropods (e.g. *Mamenchisaurus*, *Barosaurus*, *Giraffatitan*, *Sauroposeidon, Erketu*) known from the Middle-Late Jurassic and Early Cretaceous. This novelty in *Leyesaurus* shows that this feature appeared convergently in the *Leyesaurus* lineage, at the beginning of the Jurassic, and later in at least four different lineages of derived eusauropods which evolved from the Middle-Late Jurassic to the Early Cretaceous. Thus, the new taxon shows that the evolution of elongated necks is not restricted to later, more derived forms, but that it was a trend that occurred convergently in multiple lineages since the earliest phases of the evolutionary history of Sauropodomorpha.

5- *Neural arches of the cervical vertebrae with sinuous dorsal margin of the neural spine and short epipophyses—extending along two-thirds of the length of the postzygapophyses.* A distinctive characteristic present in *Leyesaurus* is the morphology of the neural spines of its cervical vertebrae. In lateral view, the anterior half of the dorsal margin of the neural spines is convex whereas the posterior half is straight to slightly concave (Figure 7). This morphology is shared with *Massospondylus* (Cooper [44], Fig. 5), but differs from that of other basal sauropodomorphs (e.g. *Riojasaurus, Plateosaurus, Yunnanosaurus, Lessemsaurus*), in which the dorsal border of the neural spines is straight and exhibits a rectangular shape in lateral view. This condition also differs from the convex dorsal borders of the cervical vertebrae present in Adeopapposaurus and *Panphagia*.

Moreover, the epipophyses of the anterior-mid cervical vertebrae of *Leyesaurus* extend along two thirds of the total length of the dorsal surface of the postzygapophyses (Figure 7), similar to the epipophyses of the cervical vertebrae of *Massospondylus* (BP/1/4934). This condition is different from *Plateosaurus*, *Yunnanosaurus, Adeopapposaurus* and *Panphagia*, in which the epipophyses almost reach the posterior margin of the postzygapophyses. It also differs from the epipophyses that extend along the entire dorsal surface of the postzygapophyses, such as present in *Pantydraco,* or from the epipophyses that overhang the rear margin of the postzygapophyses of some postaxial cervical vertebrae, such as present in the basal sauropodomorphs *Saturnalia*, *Riojasaurus*, *Melanorosaurus*, and *Lufengosaurus*; some Eusauropoda as *Omeisaurus*, *Cetiosaurus,* and *Shunosaurus;* and in Neosauropoda (sensu Yates [34]).

6- *Proximal articular surface of metatarsal III shelf-like and medially deflected.* The proximal articular surface of metatarsal III of *Leyesaurus* has a deflected medial margin that ends in a sharp border and forms a shelf-like medial margin with the convex dorsal surface and concave ventral surface (Figure 12). This medial deflection of the proximal articular surface of metatarsal III is hook-shaped (Figure 12A–B) and may overlaps the lateral margin of the proximal articular surface of metatarsal II. This condition contrasts with the straight to slightly convex medial margin of the proximal articular surface of metatarsal III that is present in all other basal sauropodomorphs (e.g. *Saturnalia*, *Riojasaurus*, *Coloradisaurus*, *Massospondylus*, *Plateosaurus*, *Unaysaurus*, *Glacialisaurus*, *Adeopapposaurus*, *Ignavusaurus*).

### Phylogenetic position

The new taxon *Leyesaurus marayensis* was added to the phylogenetic analysis of basal sauropodomorphs published by Yates [34] and later modified by Smith and Pol [45] and Yates et al. [46]. In addition, we added the recently described basal sauropodomorphs *Adeopapposaurus* [21], *Chromogisaurus* [8], S*eitaad* [47], *Ignavusaurus* [48], and *Sarahsaurus* [49] to the data matrix. The original scoring of *Coloradisaurus* made by Yates [34] and the scoring of *Adeopapposaurus* made by Sertich and Loewen [47] was modified in our data matrix (see Information S1).

**Figure 13:**
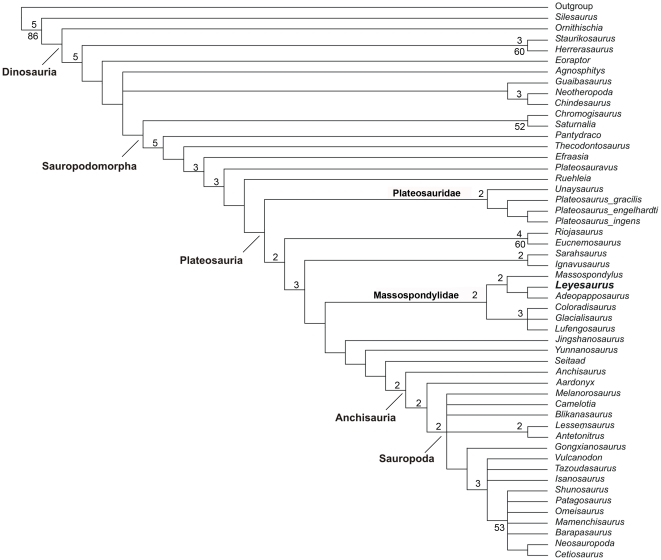
Strict consensus of the phylogenetic analysis of sauropodomorph dinosaurs. Analysis was based on the dataset of Yates [34] modified by other authors [8], [45]–[49] and including *Leyesaurus marayensis* gen. et sp. nov., showing the strict consensus of 18 MPTs. Bremer decay indices greater than 1 are listed above nodes and Bootstrap values greater than 50% are listed below nodes.

We performed an analysis using the modified dataset of 361 characters and 54 taxa usingTNT 1.1 [50], [51] with equally weighted parsimony and a heuristic search of 1000 replicates of Wagner trees followed by TBR branch swapping. 36 characters were treated as ordered, following Yates [34]. The analysis resulted in the recovery of 18 MPTs of 1301 steps each (CI: 0.325 and RI: 0.667). The strict consensus of the MPTs is shown in Figure 13.

The strict consensus tree shows a resolved placement of *Leyesaurus* within Massospondylidae (Figure 13). *Leyesaurus* is depicted as the sister-taxon of *Adeopapposaurus*, and both taxa form the sister-clade of *Massospondylus*. The *Leyesaurus*+*Adeopapposaurus* clade is supported by five unambiguous synapomorphies: transverse width of the ventral ramus of the postorbital greater than its rostrocaudal width at mid-shaft (character 53.1); jaw joint no lower than the level of the dorsal margin of the dentary (character 94.0); procumbent maxillary tooth crowns (character 110.1); postaxial cervical vertebrae with epipophyses that do not overhang the rear margin of the postzygapophyses (character 137.0); and posteromedial heel of distal tarsal four is the proximodistally deepest part of the bone (character 328.0). This clade is supported by a minimal Bremer support and has bootstrap frequencies of 49% (see Information S1 and Figure S1).

*Adeopapposaurus*+*Leyesaurus* is recovered as the sister group of *Massospondylus*, and this clade is diagnosed by six unambiguous synapomorphies: weak development of the external narial fossa (character 11.0); length of the rostral ramus of the maxilla is less than its dorsoventral depth (character 26.0); quadratojugal with its jugal ramus longer than its squamosal ramus (character 65.1); length of the first phalanx of manual digit one greater than the length of the first metacarpal (character 235.1, unknown in *Leyesaurus*); rounded to blunty pointed caudal margin of the postacetabular process of the ilium (character 258.0, unknown in *Leyesaurus*); and fourth trochanter medially located along the mediolateral axis of the femur (character 296.0, unknown in *Leyesaurus*). This group is supported by a Bremer support value of 2 and bootstrap frequencies below 50% (Figure 13, Figure S1).

The clade formed by *Massospondylus*, *Adeopapposaurus,* and *Leyesaurus* is placed as the sister group of the clade formed by *Coloradisaurus*, *Lufengosaurus* and *Glacialisaurus.* This group resembles the Massospodylidae of Yates [33], but our results imply an expanded taxonomic content for Massospondylidae, a group that is diagnosed by seven unambiguous synapomorphies: dorsal profile of the snout with a depression behind the naris (character 20.1, unknown in *Leyesaurus*); presence of a web of bone spanning junction between anterior and ventral rami of the lacrimal (character 41.1); foramina for mid-cerebral vein on occiput located on the supraoccipital (character 73.1, unknown in *Leyesaurus*); orientation of symphyseal end of the dentary strongly curved ventrally (character 99.1); elongation of the cervical vertebra 4 or 5 exceeds four times the anterior centrum height (character 131.2); laterally expanded tables at the midlength of the dorsal surface of the neural spines of the pectoral and cervical vertebrae (character 149.2, unknown in *Leyesaurus*), and symmetrical fourth trochanter of the femur (character 294.2). In this analysis, Massospondylidae is supported by a Bremer support values of 2 and bootstrap frequencies below 50% (Figure 13, Figure S1).

In this analysis, Massospondyidae and more derived sauropodomorphs share nine synapomorphies: presence of slot-shaped subnarial foramen (character 14.1); antorbital fossa on the ascending ramus of the maxilla weakly impessed and delimited by a rounded rim or a change in slope (character 31.1); crescentic antorbital fossa with strongly concave posterior margin that is roughly parallel to its anterior margin (character 32.0); length of the anterior ramus of the lacrimal less than half of the length of the ventral ramus (character 40.1); dorsal margin of the postorbital with a distinct embayment between the anterior and posterior dorsal process in lateral view (character 54.1); absence of a deep septum spanning the interbasipterygoid space (character 85.0); proximal width of the first metacarpal between 80–100 per cent of its length (character 227.2); transverse axis of the distal end of the first phalanx of manual digit one ventrolateral twisting 60 degrees relative to its proximal end (character 234.2); and presence of a notch separating the posteroventral end of the ischial obturator plate from the ischial shaft (character 268.0).

In contrast to the results presented by Sertich and Loewen [47], the taxon *Seitaad* was recovered outside Massospondylidae. All MPTs depict *Seitaad* in a more derived position than Massospondylidae, being more closely related to *Anchisaurus* and more derived sauropodomorphs (Figure 13). The synapomorphies that support *Seitaad* + Anchisauria are: length of the deltopectoral crest is between 30–50 per cent of the length of the humerus (character 207.1); and minimum transverse shaft width of first metacarpal is lesser than twice the minimum transverse shaft width of second metacarpal (character 225.0). In order to retrieve the clade formed by *Seitaad* and *Adeopapposaurus*, as was originally proposed by Sertich and Loewen [47], we have run a constrained analysis in TNT. The tree search resulted in topologies that require four extra steps than the MPTs of the unconstrained search (1305 steps, CI: 0.324 and a RI: 0.666) and a Templeton test showed that these topologies are not significantly longer than the MPTs (p = 0.1573). The strict consensus of the constrained analysis depicts *Seitaad* in a polytomy together with *Massospondylus*, *Adeopapposaurus* and *Leyesaurus*.

Similarly, *Sarahsaurus* had been initially regarded as a massospondylid by Attridge et al. [52], although the recent study of Rowe et al. [49] rejected this position. Here, *Sarahsaurus* is placed outside Massospondylidae, but in a different position than in Rowe et al. [49]. Our analysis positions *Sarahsaurus* as a plateosaurian more basal than massospondylids, and forms a clade with *Ignavusaurus* (Figure 13). The position of *Ignavusaurus* also differs from the original hypothesis given by Knoll [48] that placed this taxon as a primitive sauropodomorph. Nine synapomorphies support the *Sarahsaurus* and *Ignavusaurus* clade: no more than 14 vertebrae between cervicodorsal transition and primordial sacral vertebrae (character 146.1); parapophysis in first two dorsals located at the anterior end of the centrum (character 153.0); length of the first caudal centrum lesser than its length (character 183.1); presence of a buttress between preacetabular process and the supracetabular crest of the ilium (character 250.0); width of the conjoined pubes greater than 75 per cent of their length (character 259.1); transverse width of the distal tibia subequal to its anteroposterior length (character 306.0); anteromedial corner of the distal articular surface of the tibia forming a right angle (character 310.0); posteromedial margin of the astragalus forming a moderately sharp corner in dorsal view (character 316.0); and femoral length between 200 and 399 mm (character 353.1). Forcing *Sarahsaurus* as basal member of Sauropodomorpha implies nine extra steps (72 MPTs of 1310 steps), significantly longer by the Templeton test (p = 0.0308). In the same way, a constrained analysis with *Ignavusaurus* within basal sauropodomorphs retrieved topologies seven steps longer than the MPTs of the unconstrained search (16MPTs of 1308 steps), which are not significantly longer by the Templeton test (p = 0.1441).

In order to test the affinities of these taxa in relation to massospondylids, we have run a constrained analysis with a *Sarahsaurus* + *Ignavusaurus* clade and with *Seitaad* placed within Massospondylidae. The search retrieves 70 MPTs that are six steps longer than the MPTs of the unconstrained search (1307 steps, CI: 0.324 and RI: 0.665). The strict consensus of the constrained analysis depicts a polytomy formed by *Massospondylus*, *Seitaad*, *Coloradisaurus* (*Glacialisaurus* + *Lufengosaurus),* and *Sarahsaurus* + *Ignavusaurus* as a sister clade to the *Adeopapposaurus* + *Leyesaurus* clade. This constrained topology is not significantly longer that the MPTs (in which *Seitaad*, *Sarahsaurus,* and *Ignavusaurus* are outside of Massospondylidae), as measured by the Templeton test (p = 0.2367). Further studies on these recently described taxa are needed to assess their status and phylogenetic affinities with confidence. For further details of the Phylogenetic Analysis see Information S1 and Figure S1.

### Conclusions

The new taxon described in the present work increases our knowledge of basal sauropodomorphs, in particular our understanding of the diversity of Massospondylidae. In addition, the new taxon helps refine the age estimate of the sedimentary unit where it was found. *Leyesaurus* is the first sauropodomorph recorded from the Marayes-El Carrizal Basin that can be diagnosed by an unambiguous set of autapomorphies and combination of characters. The presence of *Leyesaurus* in the Quebrada del Barro Formation increases the possibilities of future biostratigraphic correlations of the Marayes-El Carrizal Basin with other stratigraphic units where sauropodomorphs are present (e.g. Ischigualasto-Villa Union Basin, Mogna Basin). In addition, this new evidence pulls into question the record of *Riojasaurus* from the Quebrada del Barro Formation [22], as well as the Norian age previously proposed and suggests a possible younger age for this unit.

*Leyesaurus* was a small basal sauropodomorph approximately 2.5 meters long (Figure 2) that differs from the medium to large size sauropodomorphs from the neighbor Ischigualasto-Villa Union Basin (e.g. *Riojasaurus*, *Coloradisaurus*, *Lessemsaurus*). The cranial and postcranial anatomy of *Leyesaurus* reveals phylogenetic information that indicates a close affinity to *Adeopapposaurus*, and of both as close relative of the African *Massospondylus*. The results obtained here place this group together with *Glacialisaurus*, *Coloradisaurus,* and *Lufengosaurus* within the monophyletic Massospondylidae. This clade represents a highly diverse family of which five out of six members (*Massospondylus*, *Coloradisaurus*, *Glacialisaurus*, *Adeopapposaurus*, and *Leyesaurus*) are known from the Late Triassic–Early Jurassic of the southern hemisphere, namely South America, South Africa, and Antarctica. The reinterpretation of *Coloradisaurus* as a massospondylid [33], as well as the recent discoveries of *Glacialisaurus, Adeopapposaurus,* and *Leyesaurus,* reveal that massospondylids were the most diverse family of basal sauropodomorphs from the southern hemisphere during Pangean times. The massospondylid affinities of *Lufengosaurus,* however, indicate this clade was not endemic to the southern hemisphere.

The analysis performed here rejects the massospondylid affinities of some basal sauropodomorphs (e.g. *Seitaad*, *Ignavusaurus*, *Sarahsaurus*) proposed in other studies [47]–[49] and suggests the existence of several lineages of massopodan sauropodomorphs that fall outside the three previously recognized clades Riojasauridae, Massospondylidae and Anchisauria.

## Supporting Information

Figure S1 **Strict consensus of the phylogenetic analysis of sauropodomorph dinosaurs.** Analysis was based on the dataset of Yates [34] modified by other authors [8], [45]–[49] and including *Leyesaurus marayensis* gen. et sp. nov., showing the strict consensus of 18 MPTs. Bremer decay indices are listed above the nodes and Bootstrap values are listed below the nodes.
(TIF)

Table S1 **Measurements (in millimeters) of the preserved bones of the new basal sauropodomorph** ***Leyesaurus marayensis*** **(PVL 706).** *Abbreviations*: *C3-7*, cervical vertebrae from 3 to 7; *Ca*, caudal vertebra; *dt*, distal tarsal; *mt*, metatarsal; *ph*, phalanx; *, incomplete; ∼, deformed.
(DOC)

Information S1 **1**) Character scorings changed for *Coloradisaurus brevis* in comparison with scorings provided by Yates et al. [46] (a) and Smith and Pol [45] (b). **2**) Character scorings changed for *Adeopapposaurus mognai* in comparison with scorings provided by Sertich and Loewen [47]. **3**) Scorings for all taxa analysed in the phylogenetic analyses of the modified version of the data matrix of Yates [34]. **4**) Strict consensus with Bremer support and Bootstrap (absolute frequencies) values. **5**) Complete list of synapomorphies.
(DOCX)
