# Supplementary figures and images for: A New Basal Sauropodomorph (Dinosauria: Saurischia) from Quebrada del Barro Formation (Marayes-El Carrizal Basin), Northwestern Argentina
Source: PLoS One. 2011 Nov 9;6(11):e26964. doi: 10.1371/journal.pone.0026964 (PMC3212523; doi:10.1371/journal.pone.0026964)

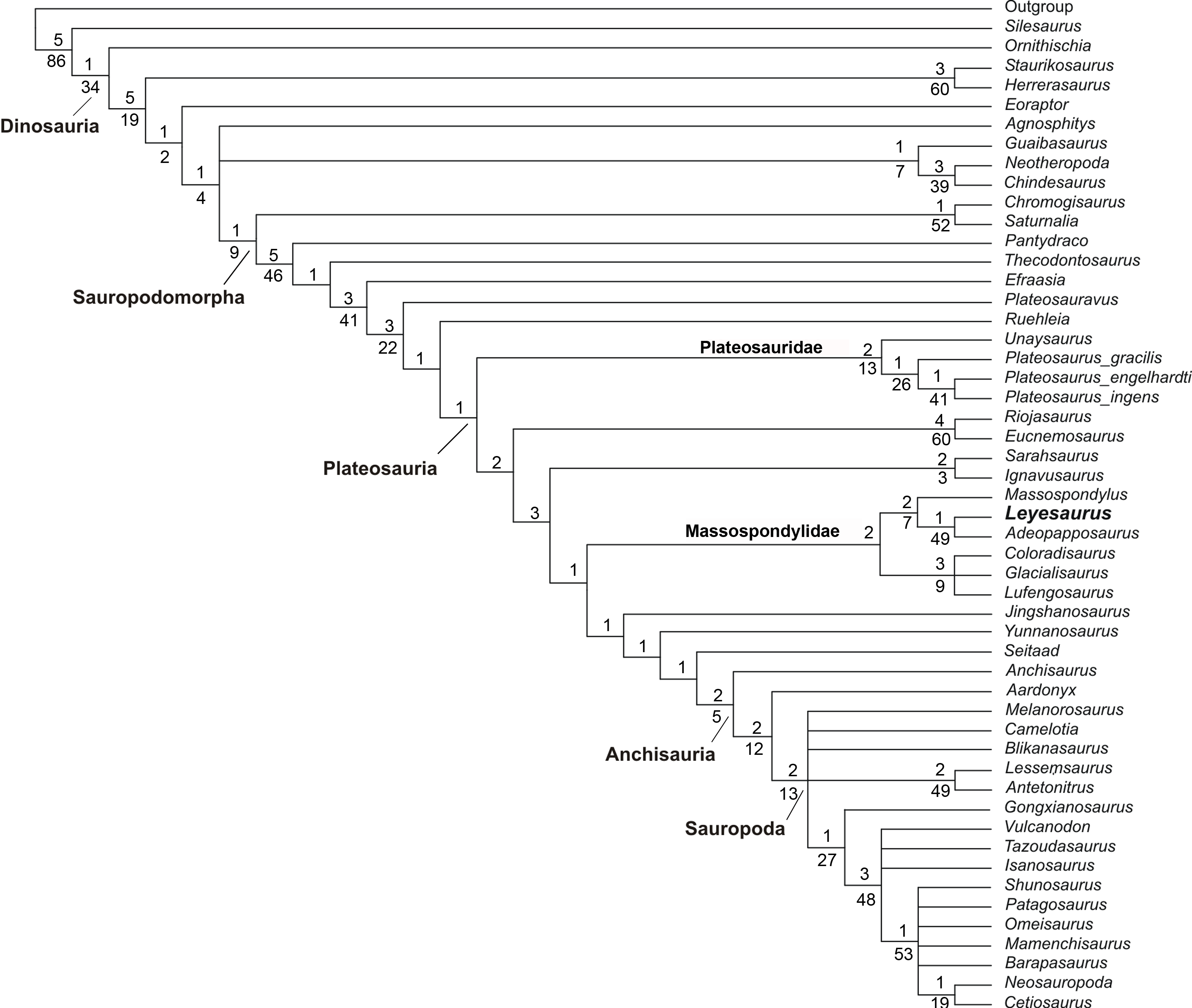

Supplement: Figure S1 — Strict consensus of the phylogenetic analysis of sauropodomorph dinosaurs. Analysis was based on the dataset of Yates [34] modified by other authors [8], [45]–[49] and including Leyesaurus marayensis gen. et sp. nov., showing the strict consensus of 18 MPTs. Bremer decay indices are listed above the nodes and Bootstrap values are listed below the nodes. (TIF) [file pone.0026964.s001.tif]
