# Supplementary material for: A New Basal Sauropodomorph (Dinosauria: Saurischia) from Quebrada del Barro Formation (Marayes-El Carrizal Basin), Northwestern Argentina
Source: PLoS One. 2011 Nov 9;6(11):e26964. doi: 10.1371/journal.pone.0026964 (PMC3212523; doi:10.1371/journal.pone.0026964)
Supplement: Table S1 — Measurements (in millimeters) of the preserved bones of the new basal sauropodomorph Leyesaurus marayensis (PVL 706). Abbreviations: C3-7, cervical vertebrae from 3 to 7; Ca, caudal vertebra; dt, distal tarsal; mt, metatarsal; ph, phalanx; *, incomplete; ∼, deformed. (DOC) [file pone.0026964.s002.doc]

**Supporting Information**

**Table S1**. **Measurements (in millimeters) of the preserved bones of the new basal sauropodomorph *Leyesaurus marayensis* (PVL 706).** Abbreviations: *C3-7*, cervical vertebrae from 3 to 7; *Ca*, caudal vertebra; *dt*, distal tarsal; *mt*, metatarsal; *ph*, phalanx; *, incomplete; ~, deformed.

| Element | Measurements of  *Leyesaurus marayensis (*PVSJ 706) in mm |
| --- | --- |
| *Skull* |  |
| Maximum anteroposterior length | 147.4 |
| Maximum dorsoventral height (anterior region to the postorbital) | 56~ |
| Maximum transverse width (frontal-parietal sutural region) | 82.7 |
| *Lower jaw* |  |
| Maximum anteroposterior length | 126* |
| Maximum antreroposterior length of the dentary | 87 |
| Maximum dorsoventral height of the dentary | 15.5 |
| *Vertebral column* |  |
| *Atlas-axis complex* |  |
| Proatlas: maximum anteroposterior length (right proatlas) | 22.5* |
| Proatlas: maximum dorsoventral height (right proatlas) | 8.8 |
| Odontoid: maximum anteroposterior length | 13.3 |
| Odontoid: maximum dorsoventral height (posterior region) | 9.5 |
| Odontoid: maximum transverse width | 13.6 |
| Intercentrum: maximum anteroposterior length | 11.7 |
| Intercentrum: maximum transverse width | 19.6 |
| Neural arch: maximum anteroposterior length | 36.4 |
| Neural arch: maximum dorsoventral height (prezygapophysis region) | 16.4* |
| Axis: maximum anteroposterior length of the centrum | 65.4~ |
| Axis: maximum anteroposterior length of the neural arch | 71.5~ |
| Axis: maximum dorsoventral height of the centrum (anterior face) | 15.9 |
| *Cervical vertebrae* |  |
| C3: maximum anteroposterior length of the centrum | 79~ |
| C3: maximum anteroposterior length of the neural arch | 107~ |
| C3: maximum dorsoventral height of the centrum (posterior face) | 20~ |
| C4: maximum anteroposterior length of the centrum | 85.1 |
| C4: maximum anteroposterior length of the neural arch | 114 |
| C4: maximum dorsoventral height of the centrum (posterior face) | 22.2 |
| C5: maximum anteroposterior length of the centrum | 97~ |
| C5: maximum anteroposterior length of the neural arch | 112~ |
| C5: maximum dorsoventral height of the centrum (anterior face) | 20~ |
| C6: maximum anteroposterior length of the centrum | 97.3 |
| C6: maximum anteroposterior length of the neural arch | 120.2 |
| C6: maximum dorsoventral height of the centrum (posterior face) | 19 |
| C7: maximum anteroposterior length of the centrum | 97.3* |
| C7: maximum anteroposterior length of the neural arch | -- |
| C7: maximum dorsoventral height of the centrum (anterior face) | 23.2~ |
| *Caudal vertebrae* |  |
| Ca ant.: maximum anteroposterior length of the centrum | 49.7~ |
| Ca ant.: maximum anteroposterior length of the neural arch | -- |
| Ca ant.: maximum dorsoventral height of the centrum (anterior face) | 38 |
| Ca ant.: maximum dorsoventral height of the centrum (posterior face) | 43.3~ |
| Ca mid.: maximum anteroposterior length of the centrum | 46.4 |
| Ca mid.: maximum anteroposterior length of the neural arch | 49* |
| Ca mid.: maximum dorsoventral height of the centrum (anterior face) | 32 |
| Ca mid.: maximum dorsoventral height of the centrum (posterior face) | 34~ |
| *Scapula* |  |
| Maximum transverse width of the glenoid region | 24.4 |
| *Humerus* |  |
| Maximum transverse width of the humeral head | 19 |
| *Pubis* |  |
| Maximum transverse width at proximal region of the apron | 44.3 |
| *Ischium* |  |
| Maximum transverse width of the iliac articular surface | 26.8 |
| Maximum dorsoventral height of the iliac articular surface | 41.7 |
| *Hindlimb*  *Distal tarsal* |  |
| dtIII: maximum transverse width | 28.3 |
| dtIII: height of the deepest region (posteromedial region) | 12.3 |
| dtIV: maximum transverse width | 30.8 |
| dtIV: height of the deepest region (posteromedial region) | 18.6 |
| *Metatarsals* |  |
| mtIII: maximum proximodistal length | -- |
| mtIII: maximum proximal height | 22.1* |
| mtIII: maximum proximal width | 32.2 |
| mtIII: maximum distal height | -- |
| mtIII: maximum distal width | -- |
| mtIV: maximum proximodistal length | 119 |
| mtIV: maximum proximal height | 15.2 |
| mtIV: maximum proximal width | 35.4 |
| mtIV: maximum distal height | 20 |
| mtIV: maximum distal width | 24.4 |
| mtV: maximum proximodistal length | 65.2 |
| mtIV: maximum proximal height | 13 |
| mtIV: maximum proximal width | 34.3 |
| mtIV: maximum distal height | 10.2 |
| mtIV: maximum distal width | 8.6 |
| *Phalanges* |  |
| ph1I: maximum proximodistal length | 48.5 |
| ph1I: maximum proximal height | 21.3 |
| ph1I: maximum proximal width | 30 |
| ph1I: maximum distal height | 16.5 |
| ph1I: maximum distal width | 22 |
| ph2II: maximum proximodistal length | 36.2 |
| ph2II: maximum proximal height | 21 |
| ph2II: maximum proximal width | 22.1 |
| ph2II: maximum distal height | 17.7 |
| ph2II: maximum distal width | 20.6 |
| ph2IV: maximum proximodistal length | 28.3* |
| ph2IV: maximum proximal height | 14.8* |
| ph2IV: maximum proximal width | 17.5 |
| ph2IV: maximum distal height | 11.8 |
| ph2IV: maximum distal width | 19.2 |
