# Supplementary material for: A New Basal Sauropodomorph (Dinosauria: Saurischia) from Quebrada del Barro Formation (Marayes-El Carrizal Basin), Northwestern Argentina
Source: PLoS One. 2011 Nov 9;6(11):e26964. doi: 10.1371/journal.pone.0026964 (PMC3212523; doi:10.1371/journal.pone.0026964)
Supplement: Information S1 — 1) Character scorings changed for Coloradisaurus brevis in comparison with scorings provided by Yates et al. [46] (a) and Smith and Pol [45] (b). 2) Character scorings changed for Adeopapposaurus mognai in comparison with scorings provided by Sertich and Loewen [47]. 3) Scorings for all taxa analysed in the phylogenetic analyses of the modified version of the data matrix of Yates [34]. 4) Strict consensus with Bremer support and Bootstrap (absolute frequencies) values. 5) Complete list of synapomorphies. (DOCX) [file pone.0026964.s003.docx]

**Information S1**

**1) Character scorings changed for *Coloradisaurus brevis* in comparison with scorings provided by Yates et al. [46] (a) and Smith and Pol [45] (b)**

(a)

31: 1→0, 43: 0→1, 45: 0→1, 48: ?→1, 55: 0→1, 57: ?→0, 71: 1→0, 78: ?→0, 79: ?→1, 104: 1→0, 107: 0→?, 108: 2→?, 109: 1→0, 120: 0→1, 123: ?→1, 126: ?→1, 137: 0→1, 176: 1→?, 177: 1→?, 178: 0→?, 180: ?→0, 182: 1→?, 190: ?→0, 191: ?→1, 200: 1→0, 201: 1→0, 204: 1→0, 209: ?→0, 230: ?→1, 245: 0→?, 247: 1→?, 249: 0→?, 267: 1→2, 272: 1→0, 273: 1→0, 279: ?→0, 282: 1→0, 294: 1→2, 304: ?→1, 312: ?→0, 330: ?→0, 340: ?→0, 344: ?→1.

(b)

355: ?→1, 356: 0→1, 359: ?→0

**2) Character scorings changed for *Adeopapposaurus mognai* in comparison with scorings provided by Sertich and Loewen [47]**

4: ?→1, 24: 0→1, 26: 1→0, 31: 0→1, 50: 0→1, 53: 0→1, 67: ?→2, 68: 1→0, 71: ?→0&1, 73: ?→1, 76: ?→0, 80: ?→1, 81: ?→0, 83: ?→0, 85: ?→0, 86: ?→1, 88: ?→1, 94: 1→0, 101: 1→0&1, 106: ?→1, 110: 0→1, 113: 0→1, 116: 1→0&1, 138: 0→1, 158: ?→1, 164: 1→0, 175: ?→0, 184: ?→0, 192: ?→1, 193: ?→0, 194: ?→0, 201: 1→0, 204: 1→0, 230: 0→1, 264: 0→1, 270: ?→1, 275: ?→0, 276: ?→1, 278: ?→0, 282: 1→0, 294: 1→2, 314: ?→0, 318: ?→1, 328: 1→0, 329: ?→1, 343: 0→1, 344: 0→1, 352: ?→0, 353: 0&1→1, 357: ?→0.

**3) Scorings for all taxa analysed in the phylogenetic analyses of the modified version of the data matrix of Yates [34].**

*Euparkeria*

00000000?0000?0000000?000?100100000000000000000?00000000010010000000000000000?0000000?00?000?000000000?1000000000000000000?00000000000???0000??000000000?00?0?00??00000000000000000000000?0?0?????????000?0020000000000??????00000000001?0?0?0000???00?0010?0000?0110000000000010000000000?02?000??01000001?000?100000?0000000??0?00010??0000??0001?100000000?000????????

*Crurotarsi*

0000000000000?0(01)000000000?0000000000(01)0000000000000000000010000000000000000000?00000000000000000000000000000000000000000000000000000000???0000??000002000?00?0?00??00002000000000000000000000000000(01)00?0(01)0?002000000000000000000000000000000000000(01)000000010?0000?0(01)00000000000000000000000?00?0000000000001?0000000000?0000000??0?000000000000000000100000000000(01)00-0??00

*Marasuchus*

00???????0?0????????????0?00???????0???????????0???????????????????????????00???00000???????????????????????00?00000000?1?100000000010???00?0??00?000000?00???00??000?00000000?000000000??01010000000?000?0121000?0000??????????????????????????????000000000000?0100010000000000000001000000011000100000000000000001000100100000?00000000000000000?00?0??00???10????????

*Aardonyx*

1101?00210002?11?11?????1110?1011000??????1010??0?11010??10?1????????1?0????????????0??????1??0?0?1?0?????1(12)?00101011001?1??????0010?110100101101?0000011?00110000000020000010?1100?0110000?01110??00?????11?????????00??00??????13101???1?10??10?????????????????1110100010011??10110?101?111100001111000???????1101110?0???01??????????010111?01?0?2??00?0???0(34)???00011

*Adeopapposaurus*

1001100210002111011111011011111010000101101000110111111111001000102001(01)01100101101010101101000001011(01)10101021111110(01)10011100111100200100011101101100200111001100000000000000100110100100000000010000000000101110001110011011110111210110011100000200001000311001000110110010?11001011000000110100001021010100000011011001001111010001110100011100120001100001100100000011

*Agnosphitys*

?0???????0?0????????????11?0?0001000????????????????????????????????????????????????????????????????????????00?000000000????????????????????????????????????????????????????????000??????????????????????????1100?00????????????????????????????????00000020000100????????????????????????????????????????????????10??????011000100??0???????????????????????????????????

*Anchisaurus*

10???00??0102?1??11?????111011?010?001010?10001100111101110?10??????01?001101?1??002110??????10?10?00??10000000101?1(12)?0111?0011???1001001111011?10000?010?001100?000??0000000??0100?0??0?01?00?10?????11(01)0??21100101100??0??1101011100100201000002000(01)10113100000?0111100010011?001010000101101000010(01)11101000000110111?1?01?010???10111?0?01??0011?0010000010011??0????1

*Antetonitrus*

?1?????????????????????????????????????????????????????????????????????????????????????????????????????????????1?1012000????????0?(01)1????????0??00?000001??00210020010121001000????????10000?01100???0?0011??31100010110??????0010?3100???1010?????????????????????011010001????????????1110111100111111110100000011011101?????????????????1011110????(12)1?00?0????31?100??1

*Barapasaurus*

???????????????????????????????????????????????????????????????????????????????????????????????????????????????1?111211???????????1?1????00??002??100101??00201101111120121100?0(12)1001?10?11??1????????101?00?1???????11?????????????????????????????11110131011000111110011???11110010???????????????????1?1?1?????????1?1???????1?????????0???????????1?1??????5????????

*Blikanasaurus* ???????????????????????????????????????????????????????????????????????????????????????????????????????????????????????????????????????????????????????????????????????????????????????????????????????????????????????????????????????????????????????????????????????????????????????????????????????????0000?0110111?1?0100101101011100101111112102110000100?211?0?1?1

*Camelotia*

???????????????????????????????????????????????????????????????????????????????????????????????????????????????????????????????????1???????????0??0??001??002100000000??00?0??????????1???0??011???????????????????????????????????????????????????????????????????1??????????1??00111?111011110111?1??110?000000????????????????????????????????????(12)???000????5????????

*Cetiosaurus*

1????????????????????????????????????????????????????????11?????????????????????????????????????????????????????????????2??0011110111110100100020?2001?10?1020??1011102010100???(12)?????1001??00100???0111101031100100011???????1?????????????????????1111013?0210?0110?1?011??1101?00???211112??0???010?111210100010010111???????????????????????????????????????5????????

*Chindesaurus*

???????????????????????????????????????????????????????????????????????????????????????????????????????????????????????????????100?????????????01?0??0?1??0?1??????000??00???????????1??0????1?00??????????????????????????????????????????????????????????0???101?1????0??????????????00?0101110000000100??00?10111100???011110100??1??????????????????????????1????????

*Chromogisaurus*

?????????????????????????????????????????????????????????????????????????????????????????????????????????????????????????????????????????????????????????????????????????????????1000??1100??1???????????????????????02?????????????????????????????0????111002202??????????????????????0??????1???10110??1000000010100010????????????????????????0??0???000???10????????

*Coloradisaurus*

?00?1002??102?111111?1??1110??011000?10??01?10?1011101110000100100?101001101101?110101?????1?1001011010011??000101010001?11?001100200100101?01100?00?001?1001100000000000000101????00?00100?001???????00001011210011?????????????????1???????????????0?0?131100???011?11002001100101110000011010000102111010000001101100??011110?00??????00011100120001100002100211111011

*Efraasia* 100?1001?010??1?111?112?1110?100???00100100000??0?10??01?0??1??????10?000?1?????0110100??????10010010??11?021001010100001???1?11001101001011011010000001??001?00000000000000000110000101000?0110000?0?010011212000111001101012010?11000001110100010000100131000101000010000001100101101000011110000101100010000001101?001?011010?0?01?1110001??0011?0010000010002??0???00

*Eoraptor*

0010?000?01010?001100000101001001?000110100000?10110010101001000001001???0??1????????????????000000?0?0?0?0?00?100000000??????100001010000?10????000??00?0????00?000??00000001??10???100??????????????01?0??011???0?0????????20?000?1?000?0?0100000110100?2?001110??0???0?0???10?0010?10????0?100??0011??000?0?10?0110??1????0???????????0?0???00?0000000000??000????????

*Eucnemosaurus*

??????????????????????????????????????????????????????????????????????????????????????????????????????????????????????????????????????????????????0???01??001100100000??00?0??????????10000?01100?????????11???????????????????????????????????????????????????????110???0?????????????001101?1010?1010110??00000110110?????????????????????????????????????????(34)????????

*Glacialisaurus*

????????????????????????????????????????????????????????????????????????????????????????????????????????????????????????????????????????????????????????????????????????????????????????????????????????????????????????????????????????????????????????????????????????????????????????0???????????????00????????????????0???1010(01)????11010111?01???????????????10111011

*Gongxianosaurus*

1?????0??????????12???????????????????????????????????????????????????????????????????????????????????????00???1??2121????????????0????????????1??0??001??0??????0?0??00000????????????0??0?0?1?0?000?011???2110????1?1?????????????????????????????0?100????0?????????????????????????(12)111?2??????0???1101??????1?0??1???0?1?1????1?11??010???10?2?1111000020005????????

*Guaibasaurus*

??????????????????????????????????????????????????????????????????????????????????????????????????????????????????????????????????????????????????0???01??011001000000??00000???????0?01100???????1????1?0??????????????????????????????????????????0?????1?00210100010000000010?10110?00001001000010?1?1010000?001111001?0?01????00101??0000??10?0000000000000110??0????

*Herrerasaurus*

00000000?0101000000000000?00010000000000100000010001010100000100001101000000101?0000000?1000?00000000?1000000000000000001010001000000100000?000010000011??0010(01)10000002000000100000101100000110010100?1110??01100000000000001200000110000011011010010000002000000000000010???01?010100100001011100010110001000010000100010010010100000011000100001000000000000?0200000000

*Ignavusaurus*

????????????????????????????????????????????????????101???????????????????????????????????????????000???????0?1101010001?????????????????????????10??001001011000000000?0000100?10000?11??0?01100?100????????????0?0?00?????????????0010??????000?00001000310001001?1011001100100101?000?0111010000101?0??1??????010100???00111010?????110???11?012?10???000?0?0(01)0000001?

*Isanosaurus*

????????????????????????????????????????????????????????????????????????????????????????????????????????????????????????????????0??0???????????2??10??0???????????????2?011000???????????????????????101?0?????????????????????????????????????????????????????????????????????????????211112??0???001?1?0??????????????????????????????????????????????????????3?110??1?

*Jingshanosaurus*

1001?002??10211111100?0?111011101100?102001??1?10111011100001000????01100000101?211001000??1?10110010??11102100101211001????111??010??????1???10100??0011100?1???0000000000000??10??01000000001000000?0010??11201011100??00??0011?3100100?010001020000100131000002011001002??110010110?01001101000010111101010000110110?12011010?00??????0101111012000100000010?4????????

*Lessemsaurus*

????????????????????????????????????????????????????????????????????????????????????????????????????????????????????????????????0??11110100??1101?0000011100210010010121000000????????????????????????0001???1110010110???????0?1?310?????1?0??1????0010013100000?01?110002???1??10110?111011?10???11111?0?000000100101???011011001??????????11??12101??00??0???311?????1

*Leyesaurus*

?001?00?1000211??11?????1010?11010000101101?00?1011111111?00100010(02)0011?????????01?10?110??1?00?10110??1010?111101000001?1??1111002001000111011????0???????????????????????????????????0000?001???000??????????????????????????????????????????????????????????????????100?????????????????????????????????????????????????????????????010???????12000????0???0??????00??

*Lufengosaurus*

100???02???0211?1?11?11111111110100101011010000101110101100010?1????0110??0110??01010100?011010010????0111?21011010100011?00011100200110111?0110100020011100110000000000000010011010010000000(01)10000000001011112(01)1011100110111101113100101201000102000010013110000201101100210110010110000101101000010211101010000110110010011110?0001?1110101110012000110000(12)(01)00410111011

*Mamenchisaurus*

11000113?11120110120000000101112??001112010101011111110121100000111221010000111?201?00?????2?11(01)0000100100011111111121113?0001110121010000110002?1110101010020110001102012100000110111111111011001011111100031100100110110011011000100011?0?10?1?31?11110131011?00111110011000110100100211112??0???010?01121010?1??0??????101011111??????1101??21?2?121111102??05?1??????

*Massospondylus*

1001100210002111111111211011111010000101101000110011011110001000102?01?011001011010101??1010010010110101010210(01)1010110011100?111002001001111011010002001110011000000000000001011101001(01)00000000000000?01(01)011112010111001101111011121001012110000020000100131000100011011001001100101110001011010000102101010000001101100100111101000111110001110012000100000210020?00?011

*Melanorosaurus*

1001?1031000211111101?1110101111???1010101101011001101011?00000010220110110010??2002010011011101?00111?1000210?10021100110?010110011011010010111?0000001??00210000000010000000?020100110010?001100????0000??21200000110??????00?0?2100101(12)0?0001020000100131000001011010001???1?010110?11101111001110011101000000110110010211010100001110010111001?101110000110?301100?11

*Neosauropoda*

1100011311112011012000001010111(02)11101112010101011(01)11110121100(01)10112221110010111120(01)200000102011100001201000(02)111111(02)12(01)112(01)00001110011110100110020121010101(01)0201110011020121100002100111001111(01)10000100111000311001(01)00111100010100000000110001??1?30011110131021000111110011001110(01)10(01)00211012??0???010?11121010?11000011111010111111011??111100210201211111021105?11???11

*Neotheropoda*

00(01)0(01)002(01)010201001100001(01)000000000000110000000010(01)100(01)01000001(01)0001(012)0100000010110000001110001000000001?00000000000000000101100110000010000(01)1000010200001010110010000001000000000(12)10001100000000010100001000101100000100010001200000010?0001001101(12)010101013000211101010000111110000110100001?011000100000000(01)011011111002001011000011111000?0?000001000000000001200000?1?

*Omeisaurus*

110001131111201101200000101011?201001112010101011111010121100000112221110010111???0?00???1???11100001?0?00021101111121113??001110120011010111002?12101010110201100011020121100?021??1?10011100100?011111100031100100011??????0100001000110001001?31011110131011000111110011000111100100211012??0???010?01021010011?0??111?011010?11??1????1111021020121111102?105????????

*Ornithischia*

0010000000000?0000000?000?1011000100100?1000000100100001000000(01)000100100000000100000000010010(01)01(01)000000100111001110100001000000000010100(01)0000??010000000?00?0?00?000000000000000(12)10000000000010000100001001111100000100000000000000000000000000000000110112100010001??000?000110000000100001?010111102000000000(01)011110002?01?(01)100000111100000000000(01)0000000000010?000????

*Pantydraco*

?0???0????????????(01)00?0???1??1????000100100000010?100001?00????????00?000?1?10??00100011?000?00?01010??0000?1001010100001001??1100?10101001101101?00???1????1???????????????????????0??11?00010000100?????11?1?00???????????????????????????????????00100121100001?????????0011??10110?00??????????1?1?000?0000000?01?001?????????????????001110011?000000001001?0??00000

*Patagosaurus*

11????????????????????????????????????????????????????????????????????????????????????????????1?00001??????0??0111012111??????1100101100000110020?2001010?00201?01111120121?00?0210011100?1?0?100???0?111?00?1100100?11?????????????????????????????1111013101000011111001100111110010?211112??0???0011111?101000??0????????????????????????????????????????????5????????

*Plateosaurus engelhardti*

1001100110102011(01)11111211110010110000100101010110011001100011001001101101101101111011000101111001011010111121001010100001100111100110100(01)01101101000200111001100000000000000101101000100000000100000000100111120001110011011110101110010020100000100001001311001020010100011111001011110000111100001011000100001011111001001101010001011100011000111001000001000400000011

*Plateosaurus gracilis*

?00??001?0102?110111?1??1110?101??00?10?1010?0??0?10001100??10??????????????????1?????????????0??0110?????12100101010?001?????1100??01???01101101?00?001?100110000000000000010?1010001010000001000?00????????1200?11100?1011?10101010000???10?????0?001001311001020010100011111001011??00001111000010110?010000?0??0110????????????????????0?????????0?0????????(23)????????

*Plateosaurus ingens*

????????????????????????????????????????????????????????????????????????????????????????????????????????????????????????????????????????????????????????????????????????????????01??01????????10???????????????????????????????????????????????????????????????????????????????????????????????????????????00?010????????????????????????????????????(01)???0?0????4????????

*Plateosauravus*

???????????????????????????????????????????????????????????????????????????????????????????????????????????????????????????????10???0100101?01101?00(12)0011?00110000000001000000??10??011???0??110????????0???(23)1110011100????????????1????????????????00100131100101?01????????11??10110?00001111000010110001000000110110??????0???????????????11?0????0??????????4????????

*Riojasaurus*

1001?00??0102011?110??00111011011000?100001000?10010?00110001000001101?0?100101?01011??????1?001000?1??101?200010101000010??1111001101001011011010001001110011000000000000001011101001000000011000??0?01001121210111100111111?011111011001010001020?0010013110010201101000110110010110010110111010110201101000000110110010011010100??011?00011100121011000001??13????????

*Ruehleia*

???????????????????????????????????????????????????????????????????????????????????????????????????????????????????????????????10???0????01???101?002001??00110010000011000000?1100001?00?0?0110??????01001111200011100?0100??01011101000???0??00???001001311000020??11000110110?10110?101011010000102100010?00?011011001?000010100?????????????????????????????3????????

*Sarahsaurus*

10?1000?10?11010?11?1?1?01101101000001000010100101110001100010?0???001000111001?110111111??1?10?10010?????011001010100001?1011110110011101110111110010010100101110000010000011111000011000010010000001000010102100111111101111011001111?01110000120210000031110102100111001111101101110211001101010101110010000010001000100011101101?1111011?110011000?1000111001??0???11

*Saturnalia*

10????????????????????????1??1????00?100?0??????????????????10?????1??00???0?0??0?0000??????????01000??????0000100010000????????0000010010??01101?00000100001100100000000000000000000101??????????????11101111100010100?????????????????????????????0??0?01100210200000000100110010010000001001100010110001000000010100010010010100000001000000001010000?000?001000000000

*Seitaad*

??????????????????????????????????????????????????????????????????????????????????????????????????????????????????????????????????????????????????00?(01)?1??00??00?001????0000?????????????????????????0000011?110001110011?11100101310?00?2110??0???0??????????????0?1000001????????????????????????????????0?00?01101?0???01101010?0??11?00?1??001???1100000010???0???0?1

*Shunosaurus*

11000113?110201101200000001011?20100111201010101001101012000011011121010001011?120020???110200100000120100011111112121112??01011?1001?1?10010012?1100001011021?0?001?0201210000?11?00?100111011001?111?1?000211001100?11000?1011001?000110000??1?3101111013101100011111?01100?111100100211112??0???000?01121010?01?0????110?101???11011??1111102002?12111?1021105????????

*Silesaurus*

00?0??00?0000?00100000??0?00?0?01010?????????0010??0????00?????????00?0?0?0000??000000?????1?00?0?010???0?0000010001000010?00000000000???0010000000000010100001100000000000000??00000100000?0?0???????111?1110000100000?????????????????????????????000000000000000000000000?010100?0?10000110110001011000100011000000000?0000000??11?1??00??000000?00???000?001100000?0?

*Staurikosaurus*

00???????????????????????????????????????????????????????????????????????????????????????????0000000001000?00?0000000000????????00010????00?01001?000011?000?00000000020000001??010101100000110010????1?1???????????????????????????????????????????000000200110000110?010?00010?00000100001??000?0101?000000001000000001????01?????????????????????????????????1????????

*Tazoudasaurus*

11??????????????????????????????????????????????????010??1?????????(02)011???1???1??????????????00?00000???0??10?1111012101???000??001010??????0002??100001??00200100011020121000????????111?1?111????11?????01?1100100??????????010?210000100?0001?310?1???03101??0?111000002??11??1??1??21100111001111011?0?0010000101010?1111011111?011??????????????1??10?1????51??0????

*Thecodontosaurus*

?0????????????????????????1??1??1000??????0?0?0?0???000????????????10?0?0?1010??011000???????00?11010??????2100101010000?1??????000101010?1?01101?000001??00110000000000000000?1000?0101100?01000??00?010011211000101001100012010001100001010000010100000121000001???0?000??011??01010?000011110000101100010000000101100??011010100????110001110?1?000??00?0????1????????

*Unaysaurus*

100110011010??1?111??1??1010?1011000????????????0??00????001???000??0?10??0?10??110100??????010?10110?????02100101010000???0?1?????0?1?0??????????0??0?11?00?10000?0??00000?????????????0????01000?00?010011?1200001100??????1????110????211???0??0??????????????????????????????????????????????????????????????110110???011010?00???????0?110???0??0???0??????1????????

*Vulcanodon*

????????????????????????????????????????????????????????????????????????????????????????????????????????????????????????????????????????????????????????????(12)????????????????????1??????0111?0?00?0?0??01???2110?10?011???????????????????????????????????310(12)?00?01111000100111110010?111011110010011?1??20?10001?0???11?1010110111011??11?01010120111110?121?05?1??00?1

*Yunnanosaurus*

100?1002??00??10011001??1011111????00101110?00??0?11011111001000001?01?0?00010???????????????10000010???0???00010?21000?1???111100110110100?0110100000011000110000000001000000?010110100000?00?0??0??001?0??11200?11100??01??0011?2101100201000(01)020?001001310000000110100010?110010111?0010110100001011010101000010011001?011010100011110010111001???11?0000110?2?0???11?

**4) Strict consensus with Bremer support and Bootstrap (absolute frequencies) values**

**Figure S1. Strict consensus of the phylogenetic analysis of sauropodomorph dinosaurs.** Analysis was based on the dataset of Yates [34] modified by other authors [8,45-49] and including *Leyesaurus marayensis* gen. et sp. nov., showing the strict consensus of 18 MPTs. Bremer decay indices are listed above the nodes and Bootstrap values are listed below the nodes.

**5) Complete list of synapomorphies**

The following list shows the unambiguous synapomorphies common to the 18 MPTs obtained in the heuristic search.

Node 57 (Eusaurischia): Character 307: 0 →1

Node 58 (*Eoraptor* + Eusaursichia): Character 18: 0→1, Character 19: 0→1, Character 25: 0→1, Character 33: 0→1, Character 38: 0→1

Node 59 (Saurischia): Character 11: 0→1, Character 13: 0→1, Character 77: 0→1, Character 127: 0→1, Character 157: 0→1, Character 205: 1→0, Character 221: 0→1, Character 222: 0→2, Character 229: 0→1, Character 238: 0→1, Character 244: 0→1, Character 321: 0→1

Node 60 (Dinosauria): Character 134: 0→1, Character 145: 0→1, Character 207: 0→1, Character 251: 0→2, Character 319: 0→1

Node 61 (*Silesaurus* + Dinosauria): Character 48: 0 →1, Character 200: 0→1, Character 203: 0→1, Character 205: 2→1, Character 259: 1→0, Character 271: 0→1, Character 284: 0→1, Character 294: 0→1, Character 304: 0→1, Character 325: 0→1, Character 327: 0→1

Node 62 (Anchisauria): Character 211: 1→0, Character 311: 0→1

Node 63 (*Seitaad* + Anchisauria): Character 207: 2→1, Character 225: 1→0

Node 64 (*Yunnanosaurus* + *Seitaad* + Anchisauria): Character 42: 0→1, Character 58: 0→1, Character 258: 2→0, Character 264: 1→0, Character 342: 0→1

Node 65 (*Jingshanosaurus* + *Yunnanosaurus* + *Seitaad* + Anchisauria): Character 21: 1→0, Character 81: 0→2, Character 173: 1→0, Character 222: 1→0, Character 253: 1→0

Node 66 (Massospondylidae + more derived sauropodomorphs): Character 14: 0→1, Character 31: 0→1, Character 32: 1→0, Character 40: 0→1, Character 54: 0→1, Character 85: 1→0, Character 227: 1→2, Character 234: 1→2, Character 268: 1→0

Node 67 ([*Sarahsaurus* + *Ignavusaurus*] + more derived sauropodomorphs): Character 50: 0→1, Character 52: 0→1, Character 132: 1→0, Character 200: 1→0, Character 264: 0→1, Character 326: 0→1, Character 353: 3→2

Node 68 (Riojasauridae + more derived sauropodomorphs): Character 29: 0→1, Character 41: 1→0, Character 57: 0→1, Character 225: 0→1, Character 242: 1→2, Character 279: 1→0, Character 296: 0→1, Character 339: 1→2

Node 69 (Plateosauria): Character 173: 0→1, Character 220: 0→1

Node 70 (*Ruehleia* + more derived sauropodomorphs): Character 205: 2→1, Character 258: 1→2

Node 71 (*Plateosauravus* + more derived sauropodomorphs): Character 149: 0 →1&2, Character 253: 0→1, Character 261: 0 →1, Character 353: 2→3

Node 72 (*Efraasia* + more derived sauropodomorphs): Character 85: 0→1, Character 94: 0→1, Character 98: 1→0, Character 131: 0→1, Character 137: 0→1, Character 177: 0→1, Character 185: 1→0, Character 191: 0→1, Character 212: 0→1, Character 227: 0→1, Character 229: 1→0, Character 244: 1→0, Character 251: 2→3, Character 306: 0→1, Character 353: 1→2

Node 73 (*Thecodontosaurus* + more derived sauropodomorphs): Character 82: 0→1, Character 97: 0→1, Character 122: 0→1

Node 74 (*Pantydraco* + more derived sauropodomorphs): Character 83: 0→1, Character 100: 0→1, Character 109: 0→1, Character 114: 0→1, Character 333: 0→1, Character 334: 0→1, Character 335: 0→1, Character 339: 0→1

Node 75 (Sauropodomorpha): Character 1: 0→1, Character 98: 0→1, Character 116: 0→1, Character 142: 0→1, Character 143: 0→1, Character158: 0→1, Character 205: 0→1, Character 211: 0→1, Character 252: 0→1, Character 304: 1→0

Node 76 (*Lessemsaurus* + *Antetonitrus*): Character 166: 0→1, Character 168: 0→1, Character 202: 0→1, Character 211: 0→1, Character 227: 2→3

Node 77 (*Melanorosaurus* + Sauropoda): Character 132: 0→1, Character 157: 1→2, Character 281: 0→1, Character 290: 0→1, Character 291: 0→1

Node 78 (*Aardonyx* + *Melanorosaurus* + Sauropoda): Character 32: 0→1, Character 167: 0→1&2, Character 234: 2→1, Character 240: 0→1, Character 280: 0→1, Character 286: 0→1, Character 353: 2→3

Node 79 (Eusauropoda): Character 298: 0→1

Node 80 (*Vulcanodon* + *Tazoudasaurus* + *Isanosaurus* + Eusauropoda): Character 187: 0→1, Character 254: 0→1, Character 315: 0→1, Character 345: 0→1

Node 81 (*Gongxianosaurus* + more derived sauropodomorphs): no autapomorphies

Node 82 (*Cetiosaurus* + Neosauropoda): Character 129: 0→1, Character 161: 0→1, Character 254: 1→2

Node 83 (*Chindesaurus* + Neotheropoda): Character 260: 0→1, Character 288: 0→1, Character 295: 1→0, Character 306: 0→1, Character 326: 0→1

Node 84 (*Guaibasaurus* + *Chindesaurus* + Neotheropoda): Character 308: 0→1, Character 318: 0→1

Node 85 (Riojasauridae): Character 283: 0→1, Character 284: 1→0, Character 289: 0→1, Character 295: 1→0

Node 86 (*Plateosaurus* *engelhardti* + *Plateosaurus ingens*): Character 353: 3→4:

Node 87 (*Plateosaurus* spp): Character 107: 0→1

Node 88 (Plateosauridae): Character 60: 0→1, Character 81: 0→1, Character 99: 0→1, Character 234: 1→2, Character 335: 1→0

Node 89 (Herrerasauridae): Character 103: 0→1, Character 151: 0→1, Character 167: 0→2, Character 180: 0→1, Character 183: 0→1, Character 189: 0→1, Character 193: 0→1, Character 199: 0→1, Character 201: 0→1, Character 265: 0→1

Node 90 (*Coloradisaurus* + *Lufengosaurus* + *Glacialisaurus*): Character 64: 0→1, Character 76: 0→1, Character 125: 1→0, Character 267: 1→2, Character 354: 0→1, Character 356: 0→1, Character 357: 0→1, Character 358: 0→1

Node 91(Massospondylidae): Character 20: 0→1, Character 41: 0→1, Character 73: 0→1, Character 99: 0→1, Character 131: 1→2, Character 149: 1→2, Character 294: 1→2

Node 92 (*Saturnalia* + *Chromogisaurus*): Character 251: 2→1, Character 288: 0→1

Node 93 (*Sarahsaurus* + *Ignavusaurus*): Character 146: 0→1, Character 153: 1→0, Character 183: 0→1, Character 250: 1→0, Character 259: 0→1, Character 306: 1→0, Character 310: 1→0, Character 316: 1→0, Character 353: 2→1

**Node 94 (*Massospondylus* + *Adeopapposaurus* + *Leyesaurus*): Character 11: 1→0, Character 26: 1→0, Character 65: 0 →1, Character 235: 0 →1, Character 258: 2→0, Character 296: 1 →0**

**Node 95 (*Adeopapposaurus* + *Leyesaurus*): Character 53: 0→1, Character 94: 1→0, Character 110: 0→1, Character 137: 1→0, Character 328: 1→0**

**Table S1**. **Measurements (in millimeters) of the preserved bones of the new basal sauropodomorph *Leyesaurus marayensis* (PVL 706).** *Abbreviations*: *C3-7*, cervical vertebrae from 3 to 7; *Ca*, caudal vertebra; *dt*, distal tarsal; *mt*, metatarsal; *ph*, phalanx; *, incomplete; ~, deformed.

| **Element** | **Measurements of**  ***Leyesaurus marayensis (*PVSJ 706) in mm** |
| --- | --- |
| *Skull* |  |
| Maximum anteroposterior length | 147.4 |
| Maximum dorsoventral height (anterior region to the postorbital) | 56~ |
| Maximum transverse width (frontal-parietal sutural region) | 82.7 |
| *Lower jaw* |  |
| Maximum anteroposterior length | 126* |
| Maximum antreroposterior length of the dentary | 87 |
| Maximum dorsoventral height of the dentary | 15.5 |
| *Vertebral column* |  |
| *Atlas-axis complex* |  |
| Proatlas: maximum anteroposterior length (right proatlas) | 22.5* |
| Proatlas: maximum dorsoventral height (right proatlas) | 8.8 |
| Odontoid: maximum anteroposterior length | 13.3 |
| Odontoid: maximum dorsoventral height (posterior region) | 9.5 |
| Odontoid: maximum transverse width | 13.6 |
| Intercentrum: maximum anteroposterior length | 11.7 |
| Intercentrum: maximum transverse width | 19.6 |
| Neural arch: maximum anteroposterior length | 36.4 |
| Neural arch: maximum dorsoventral height (prezygapophysis region) | 16.4* |
| Axis: maximum anteroposterior length of the centrum | 65.4~ |
| Axis: maximum anteroposterior length of the neural arch | 71.5~ |
| Axis: maximum dorsoventral height of the centrum (anterior face) | 15.9 |
| *Cervical vertebrae* |  |
| C3: maximum anteroposterior length of the centrum | 79~ |
| C3: maximum anteroposterior length of the neural arch | 107~ |
| C3: maximum dorsoventral height of the centrum (posterior face) | 20~ |
| C4: maximum anteroposterior length of the centrum | 85.1 |
| C4: maximum anteroposterior length of the neural arch | 114 |
| C4: maximum dorsoventral height of the centrum (posterior face) | 22.2 |
| C5: maximum anteroposterior length of the centrum | 97~ |
| C5: maximum anteroposterior length of the neural arch | 112~ |
| C5: maximum dorsoventral height of the centrum (anterior face) | 20~ |
| C6: maximum anteroposterior length of the centrum | 97.3 |
| C6: maximum anteroposterior length of the neural arch | 120.2 |
| C6: maximum dorsoventral height of the centrum (posterior face) | 19 |
| C7: maximum anteroposterior length of the centrum | 97.3* |
| C7: maximum anteroposterior length of the neural arch | -- |
| C7: maximum dorsoventral height of the centrum (anterior face) | 23.2~ |
| *Caudal vertebrae* |  |
| Ca ant.: maximum anteroposterior length of the centrum | 49.7~ |
| Ca ant.: maximum anteroposterior length of the neural arch | -- |
| Ca ant.: maximum dorsoventral height of the centrum (anterior face) | 38 |
| Ca ant.: maximum dorsoventral height of the centrum (posterior face) | 43.3~ |
| Ca mid.: maximum anteroposterior length of the centrum | 46.4 |
| Ca mid.: maximum anteroposterior length of the neural arch | 49* |
| Ca mid.: maximum dorsoventral height of the centrum (anterior face) | 32 |
| Ca mid.: maximum dorsoventral height of the centrum (posterior face) | 34~ |
| *Scapula* |  |
| Maximum transverse width of the glenoid region | 24.4 |
| *Humerus* |  |
| Maximum transverse width of the humeral head | 19 |
| *Pubis* |  |
| Maximum transverse width at proximal region of the apron | 44.3 |
| *Ischium* |  |
| Maximum transverse width of the iliac articular surface | 26.8 |
| Maximum dorsoventral height of the iliac articular surface | 41.7 |
| *Hindlimb*  *Distal tarsal* |  |
| dtIII: maximum transverse width | 28.3 |
| dtIII: height of the deepest region (posteromedial region) | 12.3 |
| dtIV: maximum transverse width | 30.8 |
| dtIV: height of the deepest region (posteromedial region) | 18.6 |
| *Metatarsals* |  |
| mtIII: maximum proximodistal length | -- |
| mtIII: maximum proximal height | 22.1* |
| mtIII: maximum proximal width | 32.2 |
| mtIII: maximum distal height | -- |
| mtIII: maximum distal width | -- |
| mtIV: maximum proximodistal length | 119 |
| mtIV: maximum proximal height | 15.2 |
| mtIV: maximum proximal width | 35.4 |
| mtIV: maximum distal height | 20 |
| mtIV: maximum distal width | 24.4 |
| mtV: maximum proximodistal length | 65.2 |
| mtIV: maximum proximal height | 13 |
| mtIV: maximum proximal width | 34.3 |
| mtIV: maximum distal height | 10.2 |
| mtIV: maximum distal width | 8.6 |
| *Phalanges* |  |
| ph1I: maximum proximodistal length | 48.5 |
| ph1I: maximum proximal height | 21.3 |
| ph1I: maximum proximal width | 30 |
| ph1I: maximum distal height | 16.5 |
| ph1I: maximum distal width | 22 |
| ph2II: maximum proximodistal length | 36.2 |
| ph2II: maximum proximal height | 21 |
| ph2II: maximum proximal width | 22.1 |
| ph2II: maximum distal height | 17.7 |
| ph2II: maximum distal width | 20.6 |
| ph2IV: maximum proximodistal length | 28.3* |
| ph2IV: maximum proximal height | 14.8* |
| ph2IV: maximum proximal width | 17.5 |
| ph2IV: maximum distal height | 11.8 |
| ph2IV: maximum distal width | 19.2 |
